# Enhancement of Spontaneous Photon Emission in Inverse Photoemission Transitions in Semiconductor Quantum Dots

**DOI:** 10.1021/acs.jpclett.3c02934

**Published:** 2024-01-04

**Authors:** Nicole Spanedda, Chandler Martin, Kevin Mesta, Arindam Chakraborty

**Affiliations:** † Department of Chemistry, Syracuse University, Syracuse, New York 13244, United States; ‡ Department of Physics, Syracuse University, Syracuse, New York 13244, United States; ¶ Department of Chemistry, Le Moyne College, Syracuse, New York 13214, United States

## Abstract

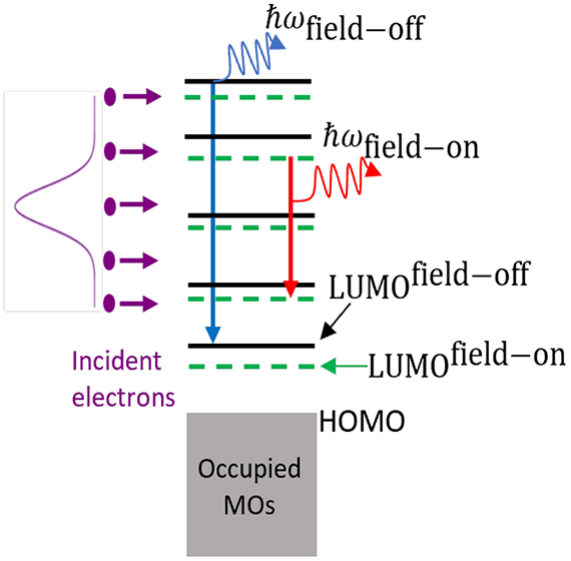

Inverse photoemission (IPE) is a radiative electron capture process where an electron is transiently captured in the conduction band (CB) followed by intraband de-excitation and spontaneous photon emission. IPE in quantum dots (QDs) bypasses optical selection rules for populating the CB and provides insights into the capacity for electron capture in the CB, the propensity for spontaneous photon emission, intraband transition energies where both initial and final states are in the CB, and the generation of photons with frequencies lower than the bandgap. Here, we demonstrate using time-dependent perturbation theory that judicious application of electric fields can significantly enhance the IPE transition in QDs. For a series of CdSe, CdS, PbSe, and PbS QDs, we present evidence of field-induced enhancement of IPE intensities (188% for Cd_54_Se_54_), field-dependent control of emitted photon frequencies (*Δω* = 0.73 eV for Cd_54_Se_54_), and enhancement of light–matter interaction using directed Stark fields (103% for Cd_54_Se_54_).

Inverse photoemission (IPE) occurs when a material captures an incident electron in one of the high-energy unoccupied states, which then can subsequently de-excite to a lower-energy unoccupied state by spontaneous emission of a photon (Figure 1). One can infer information about the unoccupied states by using the kinetic energy of the incident electron and the energy of the emitted photon. IPE, which is also referred to as radiative electron capture, has been previously used for studying electron–ion radiative recombination in electron scattering events.^1,2^ IPE spectroscopy has been used to investigate the unoccupied states of various chemical systems and materials,^3−6^ for example, the LUMO energies of various π-conjugated organic molecules and molecular organic semiconductors.^7−9^ In conjunction with other techniques, IPE spectroscopy has been used to investigate the band structure of spatially aligned graphene nanoribbons on stepped Au(788) surfaces and has also been used to probe the conduction bands of solar cell components and PbS quantum dots in thin films.^10−12^ IPE spectroscopy has also been used to investigate the effects of ion bombardment on the unoccupied electronic surface states of Ni(110).^13^ A newer technique, low-energy inverse photoemission spectroscopy (LEIPS), was developed to circumvent the damaging effects of IPE spectroscopy on organic semiconductors.^14^ It has been shown that the signal intensity for LEIPS can be enhanced by exploiting the occurrence of surface plasmon resonance in Ag nanoparticles.^15^

**Figure 1:**
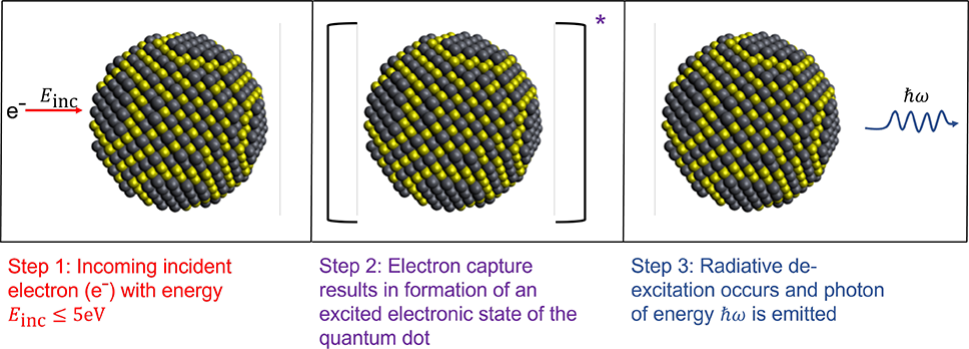
Overview of the IPE process.

The IPE process has also been theoretically and computationally investigated for some chemical systems. For example, the inverse photoemission and photoemission processes in NiO have been simulated by the use of complete active space self-consistent field theory and periodic many-body G_0_W_0_ calculations.^16^ In another work, the density of states of the valence and conduction bands of metal halide perovskites were theoretically investigated using density functional theory and experimentally investigated using IPE spectroscopy, along with ultraviolet photoemission spectroscopy.^17^ IPE spectroscopy enables the investigation of states that cannot be accessed using single-photon photoemission spectroscopy.^3^ Additionally, IPE spectroscopy is an alternative to two-photon photoemission spectroscopy and near-edge X-ray absorption fine structure (NEXAFS), which can provide direct and clear information about the unoccupied states in materials.^3,18−21^ IPE spectroscopy allows for the investigation of unoccupied states while avoiding the complications that arise from the formation of a core hole. As a result, electron–hole interactions do not need to be considered.^3,19,21^

The quantum chemical investigation of the IPE process introduces additional challenges compared with charge-neutral electronic excitations due to the unbound nature of the incoming electron. Consequently, one has to deal with not only bound states with *E* < 0 but also scattering states with *E* > 0. The computational investigation of IPE processes in quantum dots (QDs) introduces additional difficulties not encountered for small molecules. Specifically, quantum dots have a high density of states, which dramatically increase the number of possible virtual-to-virtual transitions. For example, in the case of Pb_140_S_140_, which is the largest system studied in this work, the total number of possible transitions that can take part in the IPE process is in excess of 1 million. Because of the large number of molecular orbitals and basis functions, the treatment of electron–electron correlation becomes challenging because of the computationally expensive AO-to-MO transformation of the required two-electron integrals.

Application of oriented electric field have been shown to be effective strategy not only for customizing physical and chemical properties of materials^22,23^ but also for controlling entanglement in many-electron systems.^24^ In this study, we demonstrate that the judicious selection of external static electric field strengths and directions (henceforth termed the Stark fields) can significantly enhance spontaneous photon emission from the IPE transitions. We present a new theoretical approach, developed by combining the frequency-dependent geminal-screened interaction kernel method (FD-GSIK)^25^ with time-dependent perturbation theory, for investigating IPE transitions in CdS, CdSe, PbS, and PbSe QDs. The central quantity of interest for the IPE process is the line shape function, *g*(ω) (Figure 2), and the ratio, *g*(ω_2_)/*g*(ω_1_), which can be used to quantify the relative transition probability of photon emission as a function of the emitted photon frequency (ω). The presence of a Stark field modifies the molecular orbitals and the molecular orbital energies{ϵ_*p*_^field-on^, χ_*p*_^field-on^} (Figure 3). Consequently, this changes both the QD–electron and QD–light interaction terms and modifies the ability of the QD to capture the incoming electron and spontaneously emit a photon with a given frequency. We quantify the change in the IPE transitions by defining the three critical metrics associated with the IPE line shape (*g*(ω)). The first is *I*_enhancement_ (eq 1), which quantifies the change in the most prominent transition (denoted by *g*_max_) in the IPE spectra due to application of the Stark field.
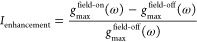
1Second, *Δω*_Stark_ quantifies the effect of the Stark field on the frequency of the dominant transition in the IPE spectra (eq 2).
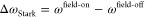
2Finally, the overall impact of the Stark fields on light–matter interactions is quantified by *Δg*_line shape_, which is defined as the cumulative change in the line shape over the frequency range [ω_1_, ω_2_] (eq 3).
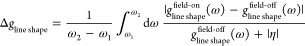
3

**Figure 2:**
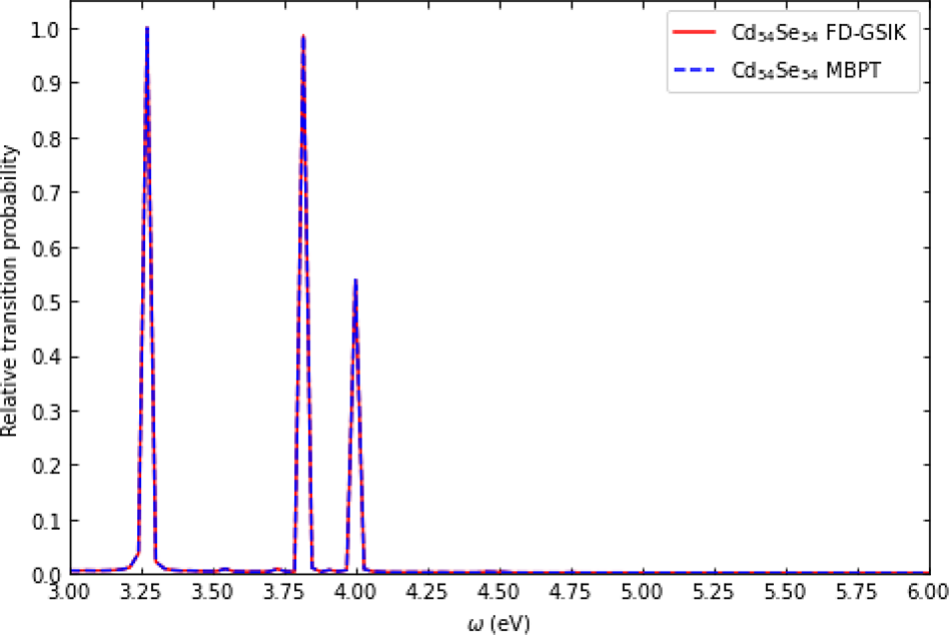
Comparison of the inverse photoemission spectra (relative transition probability as a function of emitted photon frequency) of Cd_54_Se_54_ obtained using FD-GSIK and vertex-corrected MBPT methods. The relative transition probability was obtained by dividing the transition probability by the maximum value of the transition probability.

**Figure 3:**
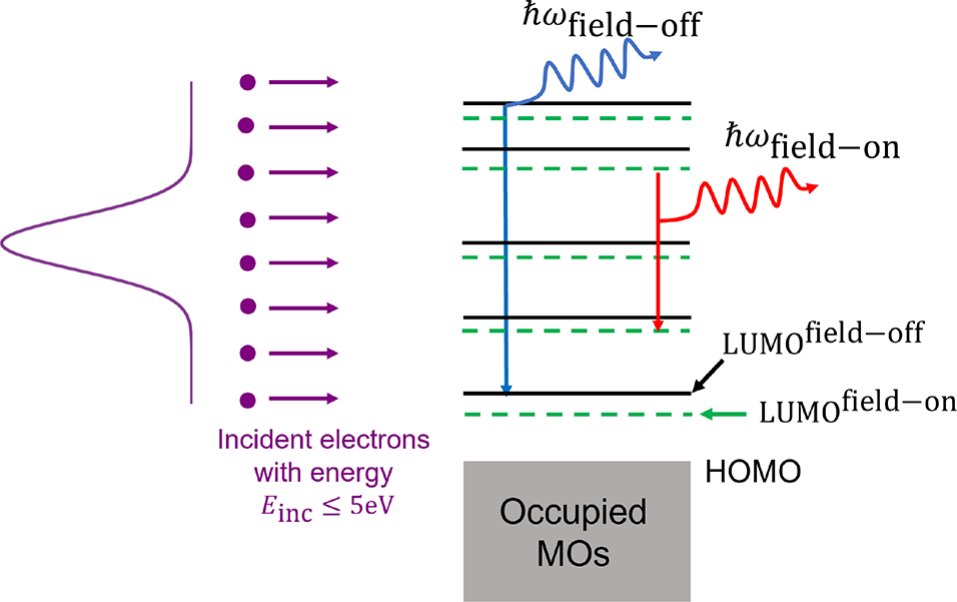
Impact of the Stark field on the overall IPE process. The incoming electrons have a distribution of incident kinetic energies (shown in purple). Application of an external electric field changes the molecular orbital energies (shown in green) and the frequency of the emitted photon (ℏω_field-on_, shown in red).

*Stark Field Increases IPE Transition Probability.* In this work, the energies of the incoming electrons, which initiated the IPE transitions, were restricted to be less than or equal to 5 eV. The impact of the external static electric field on QDs was investigated for five different electric field strengths (denoted as *E*_Stark_ = *E*_1_, ..., *E*_5_) with values [−2, −1, 0, +1, +2] × 10^–5^*E*_au_ along three Cartesian (*x*, *y*, *z*) directions. The field strengths were selected to be weak and nonionizing for all the QDs studied with *ea*_0_*E*_Stark_/*E*_IP_ ≤ 10^–5^, where *E*_au_ is the electric field in atomic units, *e* is the charge of the electron, *a*_0_ is the Bohr radius, and *E*_IP_ is the ionization potential. The *x*, *y*, and *z* components of each of these electric field vectors are provided in the Supporting Information. The percent changes in maximum relative transition probabilities for electric fields along the *x*, *y*, and *z* directions, along with the corresponding field strengths, are displayed in Table 1. For all of the four different types of QDs (CdSe, CdS, PbSe, and PbSe), the presence of the Stark fields was found to enhance the IPE transitions. The greatest enhancement of the IPE transition probabilities for Cd_24_S_24_ and Cd_45_S_45_ was achieved with an electric field aligned with these dots along the *x*-axis, while for Cd_24_Se_24_ and Cd_54_Se_54_ the greatest enhancement occurred in the presence of an electric field aligned along the *z*-axis (Table 1). Out of all of the QDs, the most significant enhancement of the IPE transition probability was observed for Pb_29_Se_29_ when the field was aligned along the *z*-axis. The Stark field also suppresses the IPE transition probability for some systems. For example, for Pb_52_Se_52_ we observed a decrease in the IPE transition probability for the fields aligned along the *y*- and *z*-axes (Table 1). A detailed analysis of the IPE spectra (for example, Cd_54_Se_54_ in Figure 4) revealed that application of a Stark field can be used to either blue-shift or red-shift the spectra with respect to the field-off case. The change in the IPE spectra for the eight QDs, listed in Table 1, is provided in the Supporting Information for five field strengths and three directions.

**Table 1:** Percent Change in Maximum Relative Transition Probabilities for Electric Fields along *x*, *y*, and *z* Directions (Eq 1)

Chemical System	*I*_enh_^*x*^	*I*_enh_^*y*^	*I*_enh_^*z*^
Cd_24_S_24_	156% (E5)	–5% (E5)	152% (E2)
Cd_45_S_45_	205% (E1)	200% (E4)	168% (E4)
Cd_24_Se_24_	33% (E2)	56% (E1)	117% (E1)
Cd_54_Se_54_	161% (E2)	35% (E1)	188% (E2)
Pb_44_S_44_	15% (E4)	15% (E4)	25% (E1)
Pb_140_S_140_	94% (E1)	19% (E1)	94% (E5)
Pb_29_Se_29_	268% (E1)	98% (E1)	279% (E1)
Pb_52_Se_52_	24% (E1)	–14% (E2)	–14% (E2)

**Figure 4:**
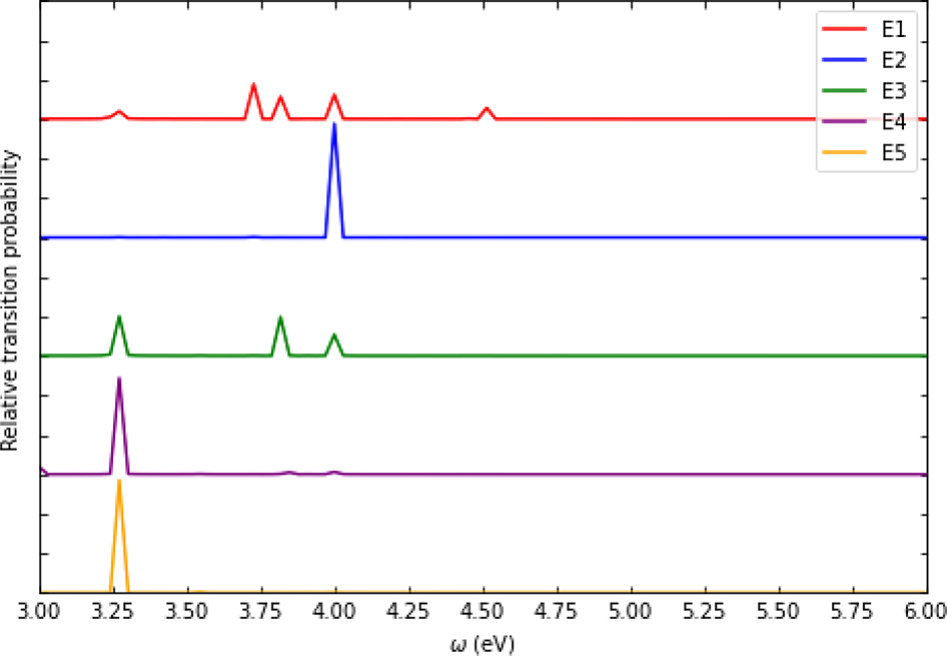
Inverse PE spectra of Cd_54_Se_54_ (relative transition probability versus ω) in the presence of electric fields (*E*_1_, *E*_2_, *E*_3_, *E*_4_, and *E*_5_) aligned with the system along the *z*-axis.

*Frequency of Spontaneously Emitted Photon Is Predominantly Blue-Shifted.* The change in the frequency of the emitted photons at maximum intensity relative to the frequency of the emitted photons for the field-off scenario (eq 2), for each field direction, is displayed in Table 2, along with the corresponding field strengths. The frequency of the emitted photons at maximum intensity depended strongly on the direction of the Stark field. For example, in the case of Cd_54_Se_54_, in the presence of the *E*_2_ field aligned along the *z*-axis, the energy of the emitted photon was found to be blue-shifted by 0.73 eV (Figure 4). However, for fields *E*_4_ and *E*_5_, no shift in the emitted frequency was observed. The Stark shift in the emitted frequency (defined in eq 2) was found to be blue-shifted for all of the QDs (Table 2) except for Cd_24_Se_24_ and Pb_29_Se_29_, both of which exhibited a red shift.

**Table 2:** Change in Emitted Photon Energies (eV) at the Maximum Relative Transition Probabilities for Electric Fields Aligned along *x*, *y*, and *z* Directions (Eq 2)

Chemical System	ω^field-off^	*Δω*_x_^field-on^	*Δω*_y_^field-on^	*Δω*_z_^field-on^
Cd_24_S_24_	3.09	0.06	0.06	0.06
Cd_45_S_45_	3.03	0.85	0.85	0.85
Cd_24_Se_24_	3.33	–0.21	–0.21	–0.30
Cd_54_Se_54_	3.27	0.55	0.73	0.73
Pb_44_S_44_	3.09	0.42	0.42	0.42
Pb_140_S_140_	3.79	0.61	0.61	0.61
Pb_29_Se_29_	0.91	–0.85	0.00	–0.73
Pb_52_Se_52_	3.21	0.18	0.15	0.15

These field-dependent IPE calculations demonstrate (Table 2) that the nonionizing weak Stark fields studied here are capable of generating a shift in the emitted photon frequency in the range [−0.8, +0.8] eV.

*Directed Stark-Field Significantly Enhances Light–Matter Interaction.* The direction of the applied Stark field can be used to enhance light–matter coupling and increase the spontaneous emission characteristic of the QDs. We demonstrated this phenomenon by calculating the cumulative change in the IPE spectra (eq 3) as a function of the Stark field direction while restricting the field strength to 10^–5^*E*_au_. A search over a set of directions was performed, and the direction corresponding to the maximum change in the IPE line shape over the entire frequency range was selected. We refer to this direction as the maximally coupled direction because this directed Stark field induced the most significant change in the light–matter interaction in the QDs.

For example, when comparing the IPE spectrum of Cd_54_Se_54_ in the presence of the maximally coupled field with the spectrum of Cd_54_Se_54_ in the absence of an external field, we found that the peak at 4.00 eV was greatly enhanced when the maximally coupled field is present (Figure 5). Additionally, there is only one dominant peak (4.00 eV) for the spectrum for Cd_54_Se_54_ in the presence of the maximally coupled field, but we see that multiple prominent peaks are observed when an external field is absent. For all the QDs, the change in the line shape along the maximally coupled field direction was found to be significantly enhanced, and *Δg* (expressed as a percentage) for nearly all of the systems were found to be greater than 100% (Table 3). The IPE spectra for Cd_24_S_24_ in the presence of the maximally coupled field displayed the greatest enhancement of light–matter coupling (193%), while Pb_44_S_44_ displayed the least enhancement (66%). The IPE spectra for all of the CdS, CdSe, PbS, and PbSe dots, in the presence of external fields aligned with these systems along maximally coupled directions, are available in the Supporting Information.

**Figure 5:**
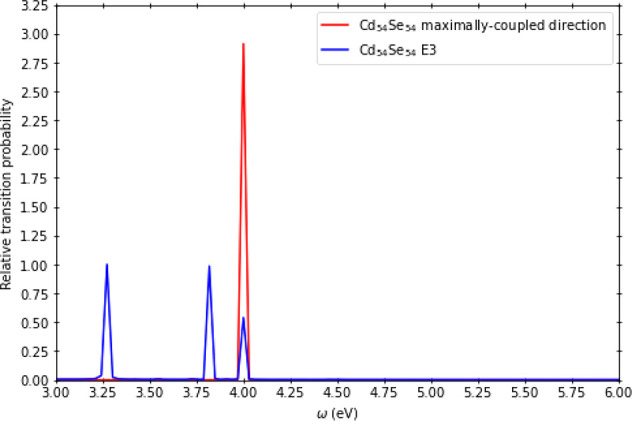
Inverse photoemission spectrum for Cd_54_Se_54_ in the presence of an electric field with which the dot is maximally coupled, along with the spectrum for the *E*_3_ field. For Cd_54_Se_54_ the maximally coupled electric field direction was found to be [−1/√2, 0, −1/√2].

**Table 3:** Overall Change in the IPE Spectra for Maximally Coupled Directed Stark Fields (Eq 3)^a^

Chemical System	*Δg*_line shape_	Optimized Stark field direction
Cd_24_S_24_ (1.3 nm)	193%	[1/√3, −1/√3, −1/√3]
Cd_45_S_45_ (1.5 nm)	189%	[0, 1, 0]
Cd_24_Se_24_ (1.3 nm)	115%	[0, −1/√2, −1/√2]
Cd_54_Se_54_ (1.8 nm)	103%	[−1/√2, 0, −1/√2]
Pb_44_S_44_ (1.5 nm)	66%	[−1/√3, −1/√3, −1/√3]
Pb_140_S_140_ (2.3 nm)	148%	[1/√3, 1/√3, −1/√3]
Pb_29_Se_29_ (1.4 nm)	155%	[−1/√3, −1/√3, 1/√3]
Pb_52_Se_52_ (1.6 nm)	189%	[1/√2, −1/√2, 0]

a The dot diameters are presented in parentheses.

*Spontaneous Photon Emission and Enhancement of Light–Matter Coupling.* As compared to photoluminescence spectroscopy, which involves both spontaneous and stimulated photon emission, IPE spectroscopy is unique because it involves only the spontaneous emission of a photon. This provides a remarkable experimental opportunity to investigate solely the spontaneous emissive characteristics of quantum dots. However, the absence of an incoming radiation field also introduces new challenges in controlling the light–matter interactions, which can otherwise be achieved by changing the intensity, power, frequency, and other optical characteristics of the incident radiation field. In this work, we demonstrated that the application of Stark fields can significantly enhance light–matter interactions and favorably impact the IPE process. Specifically, we have identified field directions that result in the greatest enhancement of light–matter interactions for CdS, CdSe, PbS, and PbSe QDs, and we have found a significant increase in the IPE transition probabilities for these systems (Table 3). These results demonstrate that the customization of IPE transition probabilities can be achieved by systematically applying Stark fields, which can be relevant for electroluminescence applications.^26^

*Accessing Unoccupied States with Low Particle-Hole Oscillator Strengths.* As opposed to absorption spectroscopy, IPE spectroscopy does not require a radiation field to induce particle-hole excitations to generate electronically excited states. Consequently, IPE can be used to investigate particle–hole excited states with low oscillator strengths that are optically dark. In conjunction with photoluminescence spectroscopic techniques, IPE spectroscopy can be used to generate enhanced maps of optically bright and dark low-lying excited states in QDs. Knowledge about excited states with low oscillator strengths can also aid in attaining greater insight into the electrochemical and cyclic voltammetric characteristics of quantum dots.^27−31^

*Photon Emission with Sub-bandgap Energies.* One of the distinguishing characteristic of the IPE process is that photon emission occurs due to unoccupied-to-unoccupied level transitions. Consequently, because of the intraband nature of the transitions, the IPE process can potentially generate photons with energies smaller than the bandgap in semiconductor materials.

*Stark Field Control of Intraband Transition.* The application of a Stark field can be used not only to increase the intensity of the IPE transition (Table 1) but also to change the frequency of the emitted photon (Table 2). This provides an exciting opportunity for narrow-bandgap materials to optimize the IPE transitions to have a strong spectral overlap with interband particle–hole transitions. We also demonstrate, for Cd_54_Se_54_, that the application of a directed Stark field can facilitate an increase in the IPE photon energy from 3.3 to 4.0 eV (Table 2).

*Stark Field Enhancement Is a Consequence of Orbital Mixing.* A zeroth-order impact of the Stark field can be understood by analyzing its effect on the uncorrelated system. The presence of the Stark field introduces a new field-dependent potential energy operator, which combines with the field-free Fock operator *F*^(*E*)^ = *F*^(*E*=0)^ + *V*^(*E*)^.
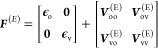
4where subscripts o and v represent the occupied and virtual blocks. The new molecular orbitals obtained from the diagonalization of the field-dependent Fock operator contain contributions from the occupied and virtual orbitals of the field-free system. As a consequence, the presence of Stark field ***V***^*E*^ induces orbital mixing,
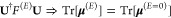
5where the elements of the unitary matrix **U** are the mixing coefficients. As a result, the field-dependent dipole-moment operator acquires contributions from virtual–virtual, occupied–occupied, and occupied–virtual blocks.
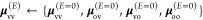
6This orbital mixing can cause transitions with low oscillator strengths to become bright and has been demonstrated in other materials.^32−37^ A detailed derivation of the field-dependent dipole-moment operator is presented in section 8 of the Supporting Information and a compact diagrammatic representation of the contributing terms to **μ**_vv_^(E)^ is presented in Figure 6.

**Figure 6:**
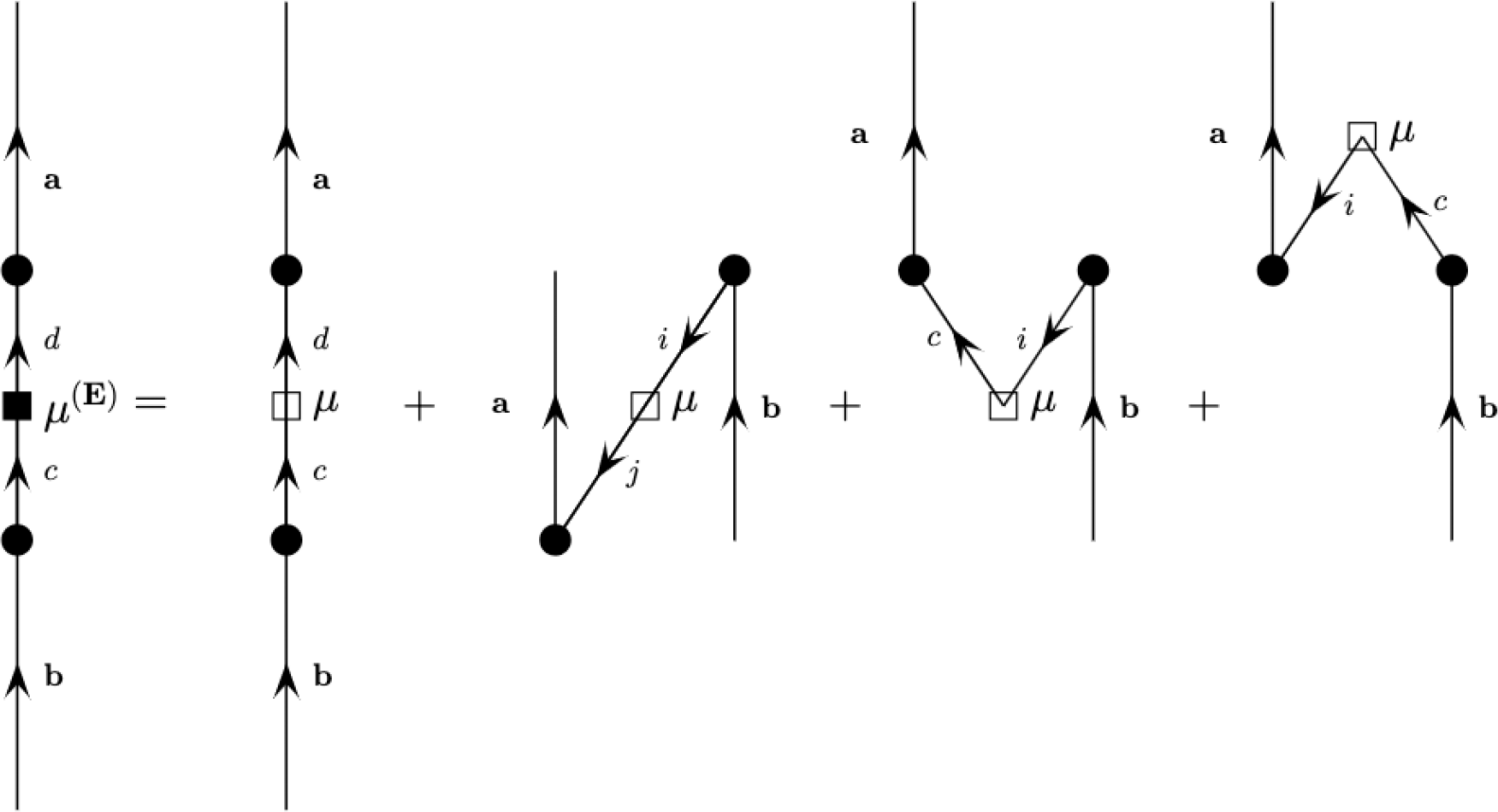
Feynman–Goldstone diagrams of the terms contributing to the field-dependent dipole-moment operator. The filled boxes and circles represent field-dependent quantities, while the unfilled boxes represent field-independent quantities.

*Calculation of Zero-Field Inverse Photoemission Spectra.* The electron capture process and subsequent spontaneous emission of the photon were calculated by using time-dependent perturbation theory (TDPT). The TDPT formulation had been derived earlier for treating radiative electron capture in electron–ion and electron–atom scattering. Using TDPT, it can be shown that up to first-order, the contribution to the IPE transition probability amplitude, *T*_**k***a*_, is given by the Feynmann–Goldstone diagram shown in Figure 7. The complete algebraic derivation of Figure 7 is presented in the Supporting Information. The incoming electron beam was described using a plane wave basis, |**k**⟩, with a distribution function of ρ_inc_(**k**) . The distribution function was constructed such that the kinetic energy of the incoming electron is uniformly distributed in the range 0–5 eV and is given by , where the proportionality constant was obtained by enforcing that ⟨ρ_inc_(**k**)⟩ = 1. The electron–QD interaction (vertex 1 in Figure 7) includes both one-body (*v*_ext_) and two-body interactions (*r*_12_^–1^ Coulomb and exchange). The field–matter interaction is treated semiclassically where the radiative de-excitation process between states |*b*⟩ and |*a*⟩ is treated using the electric dipole approximation (vertex 2 in Figure 7), and the EM field was treated as a time-dependent periodic field with frequency ω. Using Figure 7, the first-order approximation to the (|**k**⟩ → |*a*⟩) IPE transition amplitude is given in eq 7,
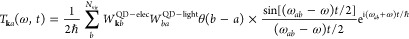
7where *W*_**k***b*_^QD–elec^ and *W*_*ab*_^QD–light^ are the coupling matrix elements for QD–electron and QD–light interactions and θ(*b* – *a*) ensures that the transitions are de-excitations. The sine term in eq 7 is the resonance contribution to transition amplitude, and the corresponding antiresonance term, with ω_*ab*_ + ω, was ignored. The line shape function, *g*(ω), was obtained by integrating over the distribution of incoming **k**-vectors and all de-excitation channels (eq 8).
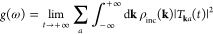
8The *t* → +∞ limit of sin(*θt*)/(*θt*) results in the delta function δ(ω_*ab*_ – ω). The IPE spectra were obtained by numerically calculating the long-time limit of eq 8, and the value of *t*_max_ was obtained by setting *t*_max_ω_*ab*_ = 1000 for the transition with the highest oscillator strength.

**Figure 7:**
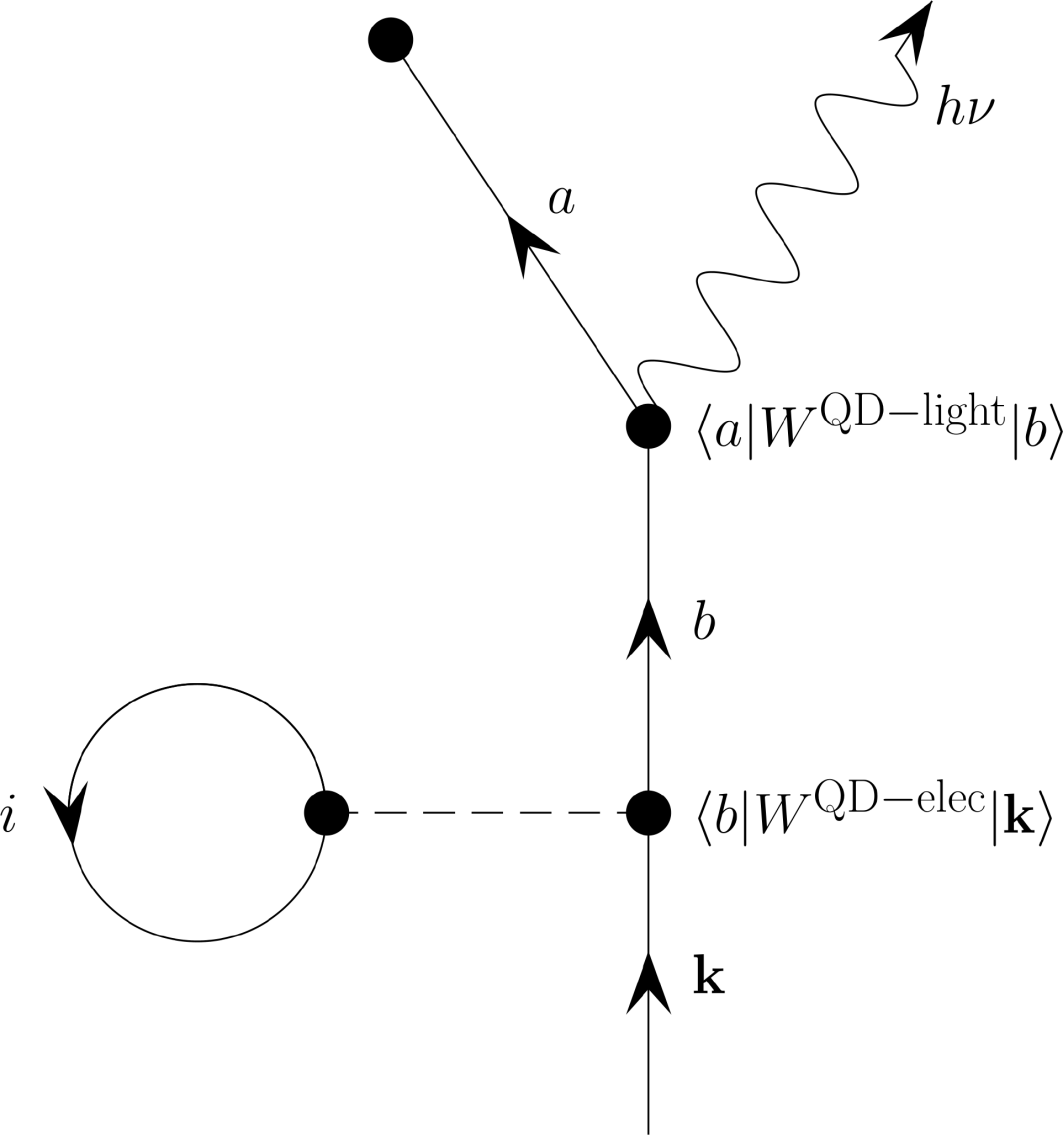
Feynman–Goldstone diagram for the first-order approximation of the inverse photoemission transition amplitude.

The first-order correction to the IPE transition amplitudes due to electron–electron correlation was obtained using the frequency-dependent geminal-screened interaction kernel (FD-GSIK) method.^25,38−40^ The FD-GSIK is a real-space method that avoids using unoccupied orbitals for constructing the electron–hole interaction kernel by performing a complete infinite-order diagrammatic summation of particle-hole excitations and deriving a renormalized real-space electron–hole correlator operator. This method also bypasses the computationally expensive AO-to-MO integral transformation step by computing all integrals directly in the real-space numerically using the stratified sampling Monte Carlo method. These two features allow the FD-GSIK method to be used for chemical systems where inclusion of a large number of unoccupied orbitals will be computationally prohibitive. In this work, FD-GSIK method was used to obtain accurate de-excitation energies ω_*ab*_^FD-GSIK^, which were then used in eq 7. In addition to FD-GSIK, electron–electron correlation effects can also be included using the vertex correction derived from MBPT.^41,42^ We compare the IPE spectrum, obtained using the FD-GSIK method, with the spectrum obtained using vertex-corrected MBPT,^41,42^ for Cd_54_Se_54_ in Figure 2. The zero-field IPE spectra for Cd_24_S_24_, Cd_45_S_45_, Cd_24_Se_24_, Cd_54_Se_54_, Pb_44_S_44_, Pb_140_S_140_, Pb_29_Se_29_, and Pb_52_Se_52_ are given in the Supporting Information. We restricted the energy of the incoming electron, which initiates the inverse photoemission event, to be less than or equal to 5 eV. We calculated the relative transition probabilities of observing the spontaneous emission of a photon in the range 3–6 eV for all of the dots. For each spectrum, the relative transition probability (ordinate) was obtained by dividing the transition probability at each value of ω (abscissa) by the maximum transition probability value. In all cases, the results from the FD-GSIK method were tested against the MBPT vertex correction method,^41^ and both methods were found to be in good agreement with each other.

The structures for the dots were obtained from their respective bulk lattices. The Hartree–Fock calculations were performed using the LANL2DZ basis with the LANL2DZ ECP potential using the TERACHEM^43−45^ electronic structure package. The integration over the plane-wave basis was performed stochastically where the incident energy *E*_inc_ was used to fix the magnitude of the **k**-vector and was sampled with 1 meV spacing in the range 0–5 eV. The direction of the **k**-vector was sampled uniformly by picking unit vectors on a unit sphere using the Marsaglia algorithm.^46,47^

*Calculation of Stark Effect on IPE Spectra.* The effect of the external DC Stark field was included as the one-body potential to the field-free Fock operator (eq 9),
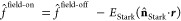
9which was diagonalized to obtain the field-dependent dressed molecular orbital and energies^48^ {ϵ_*p*_^field-on^, χ_*p*_^field-on^} which were used for calculation of the *g*^field-on^(ω) line shape function (eq 8). The direction of the Stark field was selected to be along the Cartesian directions (**n̂**_Stark_ = *x*, *y*, *z*) for a range of *E*_Stark_, and the resultant increase in the IPE transition probability is presented in Table 2. The maximally coupled electric field directions were determined by fixing the *E*_Stark_ = 1 and searching over the **n̂** directions such that *Δg*_line shape_ is maximized.
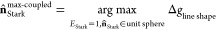
10In this work, we restricted the set of directions to be composed of 26 unique directions on the unit sphere, where each component of these vectors was restricted such that they can take on values of only −1, 0, or +1. This resulted in a set of 27 vectors, out of which the [0, 0, 0] vector was discarded and the remaining vectors were normalized to one.

## Supporting Information Available

(1) Derivation of the IPE transition probability, (2) IPE spectra for the zero-field cases, (3) effect of direction of IPE transition probability, (4) enhancement of field–matter interaction, (5) list of electric field directions, (6) diameters of the QDs, (7) HOMO–LUMO gap of the QDs, (8) derivation of Stark-field induced mixing of molecular orbitals, and (9) list of atomic coordinates of QDs (PDF)
Transparent Peer Review report available (PDF)
