# Supplementary material for: Enhancement of Spontaneous Photon Emission in Inverse Photoemission Transitions in Semiconductor Quantum Dots
Source: J Phys Chem Lett. 2024 Jan 4;15(2):364–70. doi: 10.1021/acs.jpclett.3c02934 (PMC10801682; doi:10.1021/acs.jpclett.3c02934)
Supplement: Supplementary file 1 — jz3c02934_si_001.pdf [file jz3c02934_si_001.pdf]

# Enhancement of Spontaneous Photon Emission in Inverse Photoemission Transitions in Semiconductor Quantum Dots

**Nicole Spanedda[1], Chandler Martin[2], Kevin Mesta[3], and Arindam Chakraborty[1,*]**

[1]Department of Chemistry, Syracuse University, Syracuse, New York 13244 USA
[2]Department of Physics, Syracuse University, Syracuse, New York, 13244 USA
[3]Department of Chemistry, Le Moyne College, Syracuse, New York 13214
[*]archakra@syr.edu

## ABSTRACT

This supporting information presents (1) derivation of the inverse photoemission (IPE) transition probability,(2) IPE spectra for the zero-field cases, (3) effect of Stark field direction on the IPE spectra, (4) effect of Stark field on the enhancement of field-matter interaction, (5) list of electric field directions used in this study, (6) diameters and chemical formula of the quantum dots (QDs) investigated, (7) HOMO-LUMO gap of the QDs, (8) derivation of Stark-field induced mixing of molecular orbitals, and (9) list of atomic coordinates of QDs in atomic units.

## 1 First-order propagator for spontaneous emission

$$i\hbar\frac{d}{dt}U_{\text{rad}}(t,t_0) = [H + W^{\text{rad}}(\omega,t)]U_{\text{rad}}(t,t_0) \tag{1}$$

The first-order approximation to the exact propagator in the eignbasis of $H$ ($U^{(1)}_{\text{rad}}(t)$) is known from time-dependent perturbation theory.

$$\langle\Psi_n|U^{(1)}_{\text{rad}}(t,t_0)|\Psi_m\rangle = \frac{1}{i\hbar}\int_0^t d\tau e^{i\omega_{nm}\tau/\hbar}\langle\Psi_n|W^{\text{rad}}(\omega,\tau)|\Psi_m\rangle \tag{2}$$

The light-matter interaction is treated using the electric-dipole approximation and the time-dependence of the field is treated as sine wave, as demonstrated in Equation 3.

$$\langle\Psi_n|W^{\text{rad}}(\tau,\omega)|\Psi_m\rangle = \frac{1}{2i}W^{\text{dipole}}_{nm}(e^{i\omega\tau} - e^{-i\omega\tau}) \tag{3}$$

Substituting Equation 3 into Equation 2, the first-order propagator can be expressed as,

$$\langle\Psi_n|U^{(1)}_{\text{rad}}(t,t_0)|\Psi_m\rangle = \frac{-W^{\text{dipole}}_{nm}}{2\hbar}\int_0^t d\tau e^{i(\omega_{nm}+\omega)\tau/\hbar} - e^{i(\omega_{nm}-\omega)\tau/\hbar} \tag{4}$$

and after integration we obtain,

$$\langle\Psi_n|U^{(1)}_{\text{rad}}(t,t_0)|\Psi_m\rangle = \frac{W^{\text{dipole}}_{nm}}{2i\hbar}\left[\frac{1-e^{i(\omega_{nm}+\omega)t/\hbar}}{\omega_{nm}+\omega} - \frac{1-e^{i(\omega_{nm}-\omega)t/\hbar}}{\omega_{nm}-\omega}\right] \tag{5}$$

which can then be simplified to the sine function line-shape as follows.

$$\langle\Psi_n|U^{(1)}_{\text{rad}}(t,t_0)|\Psi_m\rangle = \frac{W^{\text{dipole}}_{nm}}{2i\hbar}\left[ie^{i(\omega_{nm}+\omega)t/\hbar}\frac{\sin[(\omega_{nm}+\omega)t/2]}{(\omega_{nm}+\omega)/2} - ie^{i(\omega_{nm}+\omega)t/\hbar}\frac{\sin[(\omega_{nm}-\omega)t/2]}{(\omega_{nm}-\omega)/2}\right] \tag{6}$$

$$\langle\Psi_n|U^{(1)}_{\text{rad}}(t,t_0)|\Psi_m\rangle = \frac{W^{\text{dipole}}_{nm}}{2\hbar}\frac{\sin[(\omega_{nm}-\omega)t/2]}{(\omega_{nm}-\omega)/2}e^{i(\omega_{nm}+\omega)t/\hbar} \tag{7}$$

The final result shows that,

$$|\Psi(t)\rangle = U_{\rm rad}(t,0)|\Psi_{\rm in}\rangle \tag{8}$$

and the probability of transition to the final state is given as

$$P_f(t) = |\langle\Psi_f|\Psi(t)\rangle|^2 \tag{9}$$

We define the transition rate per unit time $\Gamma_f$ as,

$$P_f(t) = t\Gamma_f \tag{10}$$

where $\Gamma_f$ is obtained using numerical approximation to first-order derivative,

$$\Gamma_f = \frac{P_f(t+\Delta t)}{\Delta t} \tag{11}$$

The initial state preparation is defined as:

$$|\Psi_{\rm in}\rangle = \Omega_+|\Psi_0^N\rangle \tag{12}$$

The expression for the Möller operator in the eigenket of H is given as

$$\langle\Psi_n^{N+1}|\Omega_+|\Psi_0^N\rangle = \sum_b \int_{-\infty}^{+\infty} d\mathbf{k}\rho_{\rm inc}(\mathbf{k})\langle\mathbf{k}|w^{\rm e-dot}|b\rangle\langle\Psi_n^{N+1}|b^\dagger|\Psi_0^N\rangle \tag{13}$$

The probability density $\rho_{\rm inc}(\mathbf{k})$ is defined as,

$$\rho_{\rm inc}(\mathbf{k}) = C\theta(E_{\rm inc} - \frac{\hbar^2k^2}{2m}) \tag{14}$$

and the proportionality constant $C$ is obtained from the normalization condition. Integrating over k-space we get,

$$C^{-1} = \int_{-\infty}^{+\infty} d\mathbf{k}\theta(E_{\rm inc} - \frac{\hbar^2k^2}{2m}) \tag{15}$$

Transforming into spherical polar coordinates and integrating over the angular coordinates,

$$C^{-1} = 4\pi\int_0^{+\infty} dkk^2\theta(E_{\rm inc} - \frac{\hbar^2k^2}{2m}) \tag{16}$$

which can be written as,

$$C^{-1} = 4\pi\int_0^{k_{\rm max}} dkk^2 = \frac{4}{3}\pi k_{\rm max}^3 \tag{17}$$

where,

$$E_{\rm inc} = \frac{\hbar^2k_{\rm max}^2}{2m} \tag{18}$$

The interaction between the incoming electron and the QD is given by,

$$\langle\mathbf{k}|w^{\rm e-dot}|b\rangle = \langle\mathbf{k}|v^{\rm ext}|b\rangle + \sum_{i=1}^N\langle\mathbf{k}i|r_{12}^{-1}(1-P_{12})|bi\rangle \tag{19}$$

Substituting,

$$\langle\Psi_f|\Psi(t)\rangle = \langle\Psi_f|U_{\rm rad}(t,0)\Omega_+|\Psi_0^N\rangle \tag{20}$$

Inserting a complete set of eigenkets of the $H$,

$$\langle\Psi_f|\Psi(t)\rangle = \sum_m\langle\Psi_f|U_{\rm rad}(t,\omega)|\Psi_m\rangle\langle\Psi_m|\Omega_+|\Psi_0^N\rangle \tag{21}$$

which implies,

$$\langle \Psi_f | \Psi(t) \rangle = \sum_b \sum_m \int_{-\infty}^{+\infty} d\mathbf{k} \theta(E_{\text{inc}} - \frac{\hbar^2 k^2}{2m}) \langle \Psi_f | U_{\text{rad}}(t, \omega) | \Psi_m \rangle \langle \mathbf{k} | w^{\text{e-dot}} | b \rangle \langle \Psi_m^{N+1} | b^\dagger | \Psi_0^N \rangle \tag{22}$$

Defining the pole-strength $A_{mb}$ as,

$$A_{mb} = \langle \Psi_m^{N+1} | b^\dagger | \Psi_0^N \rangle \tag{23}$$

and the matrix element $w_{\mathbf{k}b}^{\text{e-dot}}$ as,

$$w_{\mathbf{k}b}^{\text{e-dot}} = \langle \mathbf{k} | w^{\text{e-dot}} | b \rangle \tag{24}$$

The probability amplitude can be expressed as follows.

$$\langle \Psi_a | \Psi(t) \rangle = \frac{1}{2\hbar} \sum_b \sum_m \int_{-\infty}^{+\infty} d\mathbf{k} \rho_{\text{inc}}(\mathbf{k}) W_{am}^{\text{dipole}} w_{\mathbf{k}b}^{\text{e-dot}} A_{mb} \frac{\sin[(\omega_{am} - \omega)t/2]}{(\omega_{am} - \omega)/2} e^{i(\omega_{nm} + \omega)t/\hbar} \tag{25}$$

## 2 Zero-field inverse photoemission spectra

In subplot A of Figure 1, we see that there are two prominent peaks of the inverse PE spectra of $Cd_{24}S_{24}$, while in subplot B we see that there are four prominent peaks of the Inverse PE spectrum of $Cd_{45}S_{45}$. The peak with the greatest intensity for $Cd_{24}S_{24}$ occurs at 3.091 eV. The second most prominent peak of this spectrum occurs at approximately 3.65 eV. The peak with the greatest intensity for $Cd_{45}S_{45}$ occurs at 3.030 eV. The second largest peak of this spectrum occurs at approximately 3.85 eV while the third and fourth largest peaks occur at approximately 3.65 and 3.25 eV, respectively. As can be observed from interpreting Figure 1, the most probable energy of the emitted photon, due to the occurrence of an inverse PE event, is very close to 3 eV for both of the CdS dots. In subplot A of Figure 2, we see that there are four prominent peaks of the inverse PE

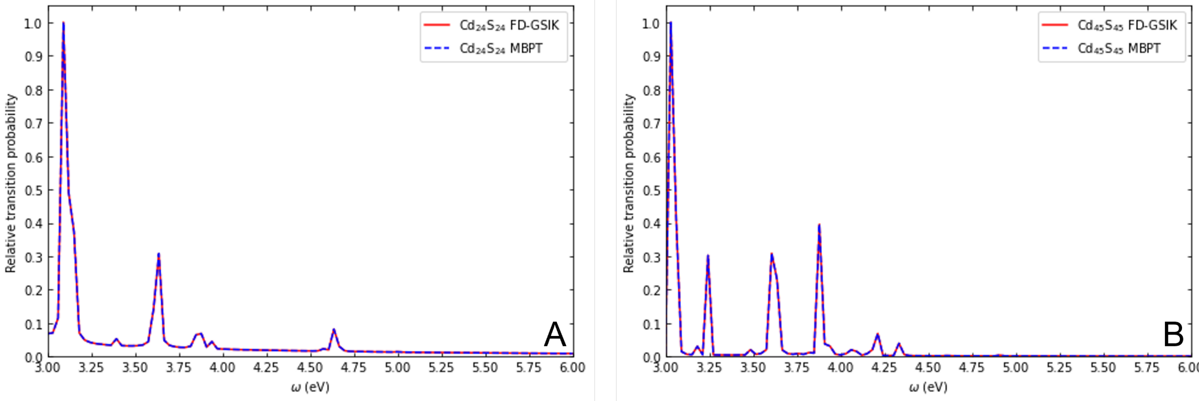


**Figure 1.** Subplots A and B (relative transition probability versus $\omega$) display the inverse photoemission spectra obtained using the FD-GSIK method and MBPT for the $Cd_{24}S_{24}$ and $Cd_{45}S_{45}$ quantum dot, respectively.

spectra of $Cd_{24}Se_{24}$, while in subplot B we see that there are three prominent peaks of the Inverse PE spectrum of $Cd_{54}Se_{54}$. The peak with the greatest intensity for $Cd_{24}Se_{24}$ occurs at 3.333 eV. The second most prominent peak of this spectrum occurs at approximately 3.15 eV, while the third and fourth largest peaks occur at approximately 4.50 and 3.50 eV, respectively. The peak with the greatest intensity for $Cd_{54}Se_{54}$ occurs at 3.273 eV. The second and third largest peaks of this spectrum occur at approximately 3.80 eV and 4.00 eV, respectively. In subplot A of Figure 3, we see that there are four prominent peaks of the inverse PE spectra of $Pb_{44}S_{44}$, while in subplot B we see that there are six prominent peaks of the Inverse PE spectrum of $Pb_{140}S_{140}$. The peak with the greatest intensity for $Pb_{44}S_{44}$ occurs at 3.091 eV. The second most prominent peak of this spectrum occurs at approximately 3.40 eV, while the third and fourth largest peaks occur at approximately 3.50 and 3.25 eV, respectively. The peak with the greatest intensity for $Pb_{140}S_{140}$ occurs at 3.788 eV. The second and third largest peaks of this spectrum occur at approximately 3.65 eV and 3.20 eV, respectively, while the fourth, fifth, and sixth largest peaks occur at approximately 4.15 eV, 4.10 eV and 3.05 eV, respectively In subplot A of Figure 4, we see that there are five prominent

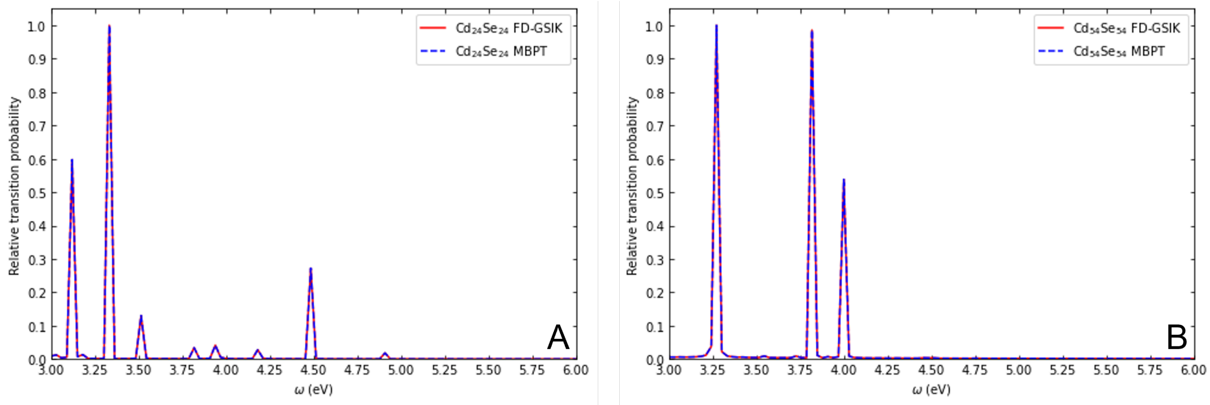


**Figure 2.** Subplots A and B (relative transition probability versus $\omega$) display the inverse photoemission spectra obtained using the FD-GSIK method and MBPT for the $Cd_{24}Se_{24}$ and $Cd_{54}Se_{54}$ quantum dots, respectively.

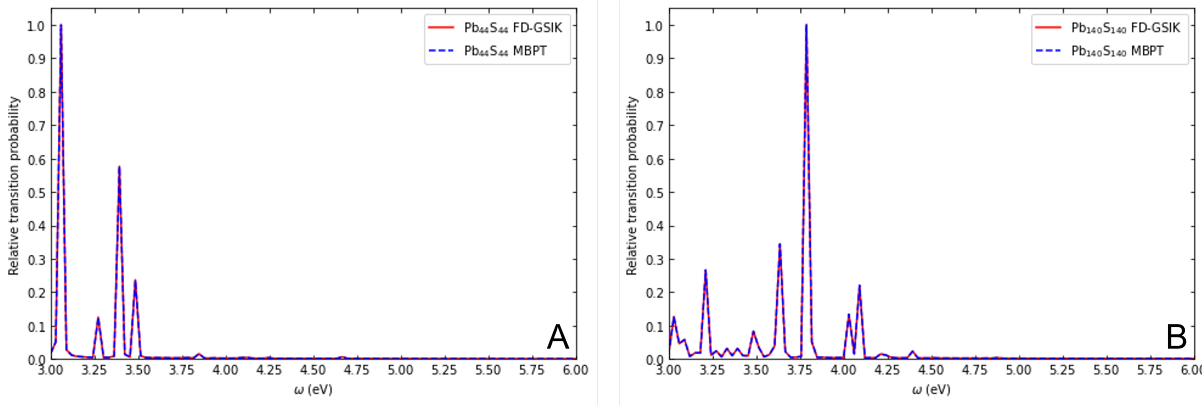


**Figure 3.** Subplots A and B (relative transition probability versus $\omega$) display the inverse photoemission spectra obtained using the FD-GSIK method and MBPT for the $Pb_{44}S_{44}$ and $Pb_{140}S_{140}$ quantum dots, respectively.

peaks of the inverse PE spectra of $Pb_{29}Se_{29}$, while in subplot B we see that there are two prominent peaks of the Inverse PE spectrum of $Pb_{52}Se_{52}$. The peak with the greatest intensity for $Pb_{29}Se_{29}$ occurs at 0.909 eV. The second and third largest peaks of this spectrum occur at approximately 1.10 eV and 0.60 eV, respectively, while the fourth and fifth largest peaks occur at approximately 0.10 eV and 2.00 eV, respectively. The peak with the greatest intensity for $Pb_{52}Se_{52}$ occurs at 3.212 eV, while the second most prominent peak of this spectrum occurs at approximately 3.15 eV .

## 3 Effect of direction of IPE transition probability

Figures 5-12 contain plots of the relative transition probability (ordinate) versus $\omega$ (abscissa) for CdS, CdSe, PbS, and PbSe quantum dots, in the presence of electric fields of differing strengths (E1, E2, E3, E4, and E5). For each spectrum in figures 5-12, the relative transition probability was obtained by dividing the transition probability at each value of $\omega$ by the maximum value of the transition probability for the E3 spectrum. It is relevant to note that the spectrum labeled E3, was obtained for the quantum dots in the absence of an electric field. For the plots subplots A in each figure, the electric fields are aligned with the quantum dots along the x-direction. For subplots B and C, the electric fields are aligned with the quantum dots along the y-direction and z-direction, respectively.

For each electric field, the value of $\omega$ at which the maximum relative transition probability occurs corresponds to the most probable energy of the photon that is emitted as a result of an inverse photoemission event, under the conditions previously described. In order to effectively visualize the spectra for all of the electric fields on a single plot for each direction, we shifted the values of the relative transition probabilities for each spectrum by adding a unique constant. The E1 spectrum was shifted

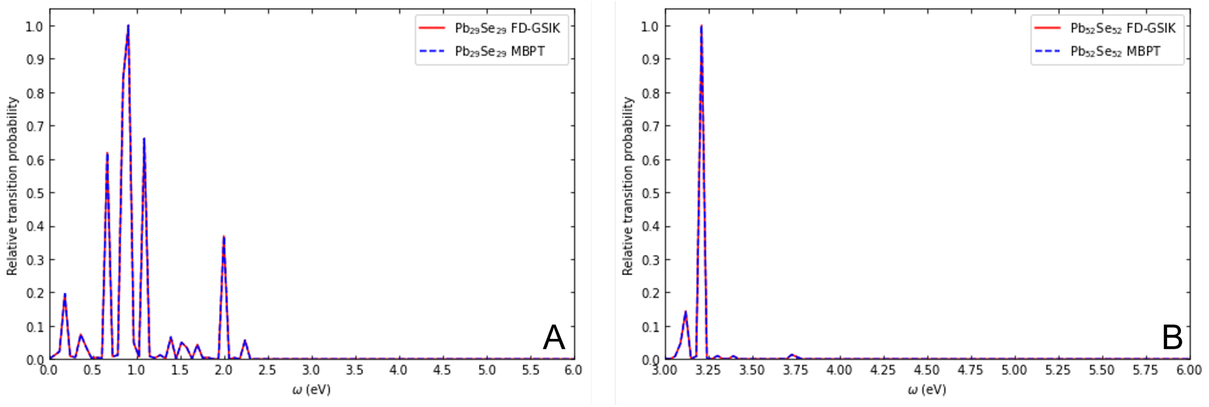


**Figure 4.** Subplots A and B (relative transition probability versus $\omega$) display the inverse photoemission spectra obtained using the FD-GSIK method and MBPT for the $Pb_{29}Se_{29}$ and $Pb_{52}Se_{52}$ quantum dots, respectively.

upwards by adding 12 to each value of the relative transition probability and the E2 spectrum was shifted upwards by adding 9 to each value of the relative transition probability. The E3 and E4 spectra were shifted upwards by adding 6 and 3 to each value of the relative transition probabilities, respectively. The values of the relative transition probability were not shifted for the E5 spectrum. section 5 in the supplementary information provides descriptions of the electric fields, E1, E2, E3, E4, and E5.

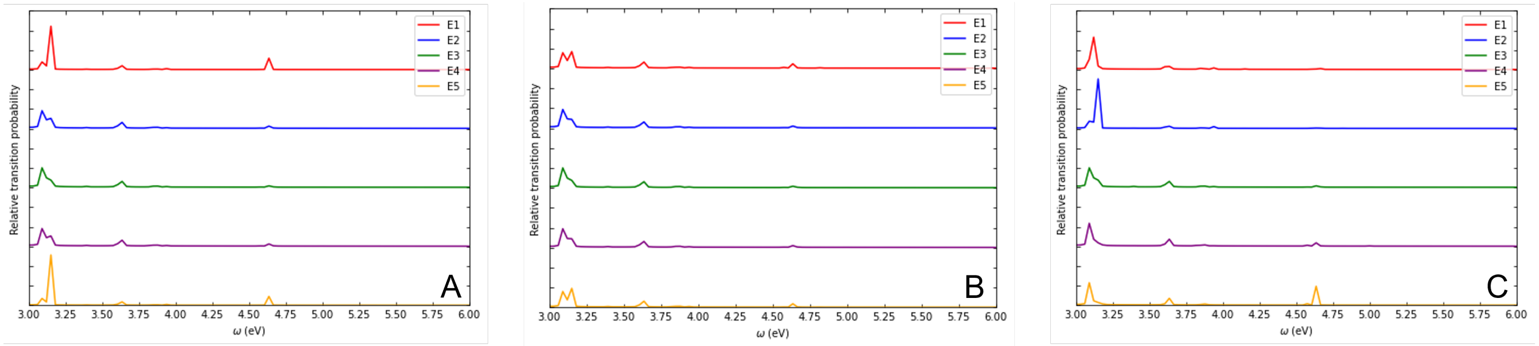


**Figure 5.** This figure contains plots of the relative transition probability (ordinate) versus $\omega$ (abscissa) for a $Cd_{24}S_{24}$ quantum dot in the presence of electric fields of differing strengths (E1, E2, E3, E4, and E5). Subplot A includes the spectra for when the electric fields are aligned with the quantum dot along the x-direction. Subplots B and C include the spectra for when the electric fields are aligned with the dot along the y-direction and z-direction, respectively.

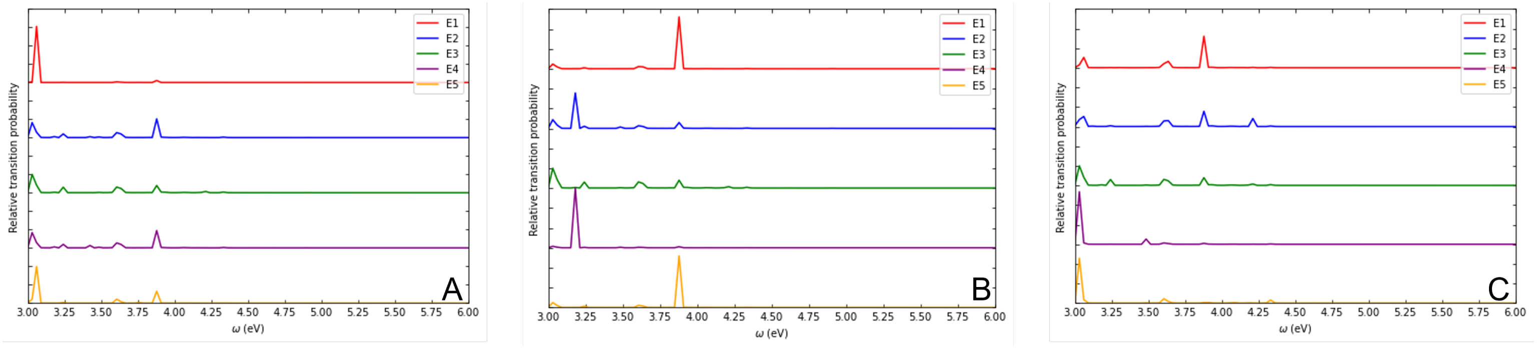


**Figure 6.** In this figure, plots of the relative transition probability (ordinate) versus $\omega$ (abscissa), for a $Cd_{45}S_{45}$ quantum dot in the presence of electric fields of differing strengths (E1, E2, E3, E4, and E5) are displayed. Subplot A includes the spectra for when the electric fields are aligned with the quantum dot along the x-direction. Subplots B and C include the spectra for when the electric fields are aligned with the dot along the y-direction and z-direction, respectively.

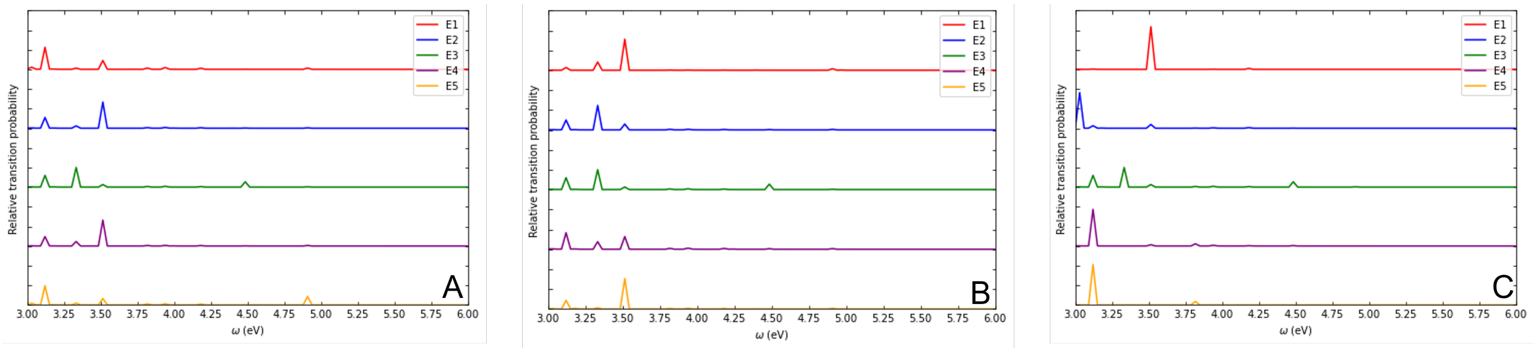


**Figure 7.** Here, we display plots of the relative transition probability (ordinate) versus $\omega$ (abscissa) for a $Cd_{24}Se_{24}$ quantum dot, in the presence of electric fields of differing strengths (E1, E2, E3, E4, and E5). Subplot A includes the spectra for when the electric fields are aligned with the quantum dot along the x-direction. Subplots B and C include the spectra for when the electric fields are aligned with the dot along the y-direction and z-direction, respectively.

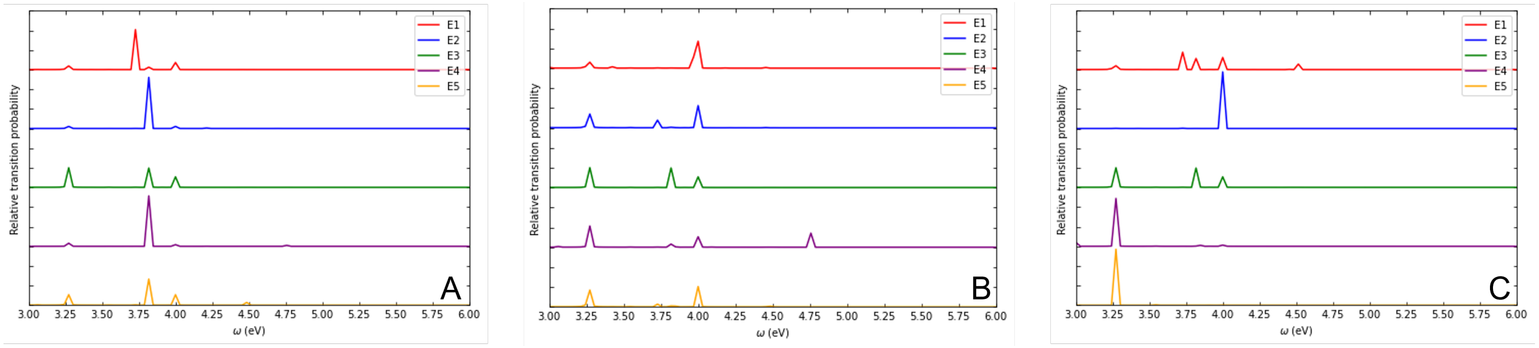


**Figure 8.** This figure contains plots of the relative transition probability (ordinate) versus $\omega$ (abscissa) for a $Cd_{54}Se_{54}$ quantum dot in the presence of electric fields of differing strengths (E1, E2, E3, E4, and E5). Subplot A includes the spectra for when the electric fields are aligned with the quantum dot along the x-direction. Subplots B and C include the spectra for when the electric fields are aligned with the dot along the y-direction and z-direction, respectively.

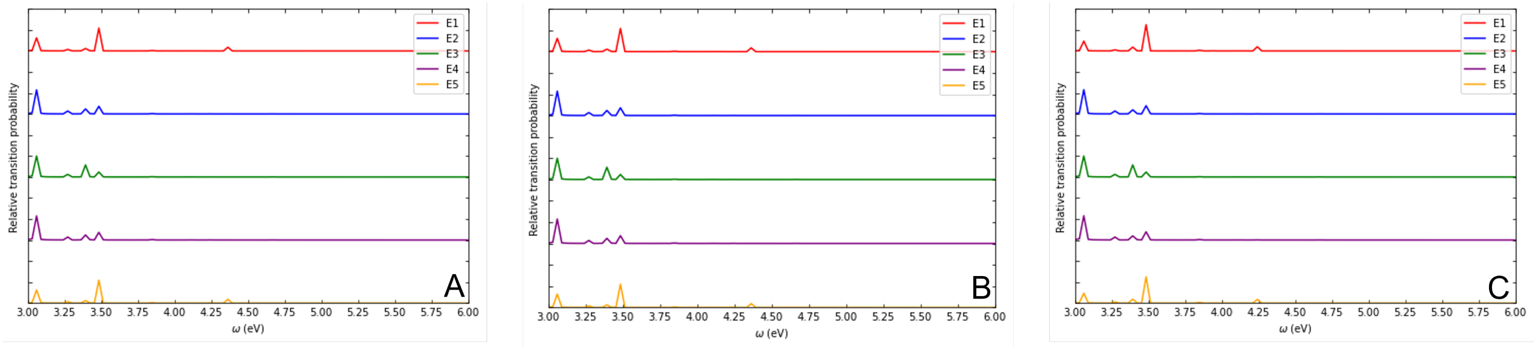


**Figure 9.** In this figure, plots of the relative transition probability (ordinate) versus $\omega$ (abscissa), for a $Pb_{44}S_{44}$ quantum dot in the presence of electric fields of differing strengths (E1, E2, E3, E4, and E5) are displayed. Subplot A includes the spectra for when the electric fields are aligned with the quantum dot along the x-direction. Subplots B and C include the spectra for when the electric fields are aligned with the dot along the y-direction and z-direction, respectively.

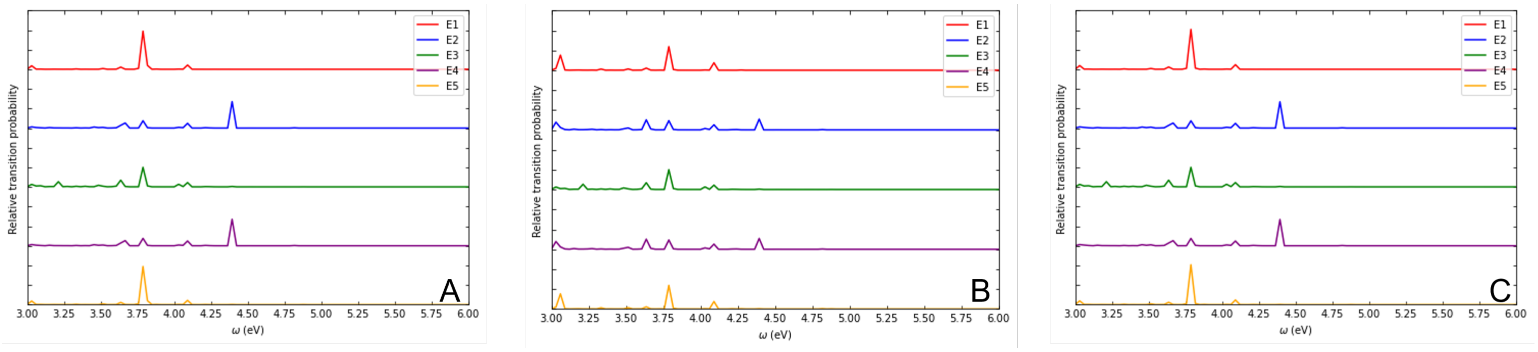


**Figure 10.** Here, we display plots of the relative transition probability (ordinate) versus $\omega$ (abscissa) for a $Pb_{140}S_{140}$ quantum dot, in the presence of electric fields of differing strengths (E1, E2, E3, E4, and E5). Subplot A includes the spectra for when the electric fields are aligned with the quantum dot along the x-direction. Subplots B and C include the spectra for when the electric fields are aligned with the dot along the y-direction and z-direction, respectively.

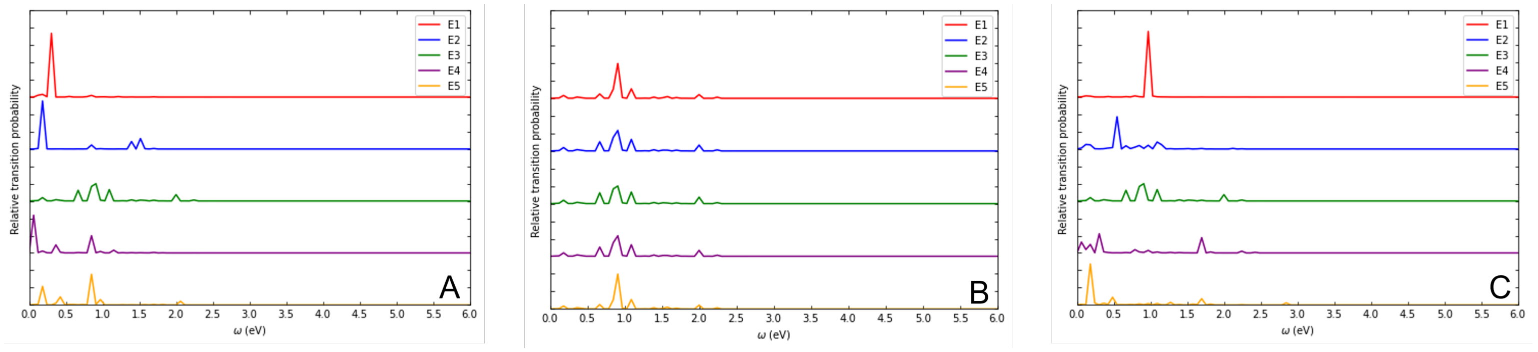


**Figure 11.** In this figure, plots of the relative transition probability (ordinate) versus $\omega$ (abscissa), for a $Pb_{29}Se_{29}$ quantum dot in the presence of electric fields of differing strengths (E1, E2, E3, E4, and E5) are displayed. Subplot A includes the spectra for when the electric fields are aligned with the quantum dot along the x-direction. Subplots B and C include the spectra for when the electric fields are aligned with the dot along the y-direction and z-direction, respectively.

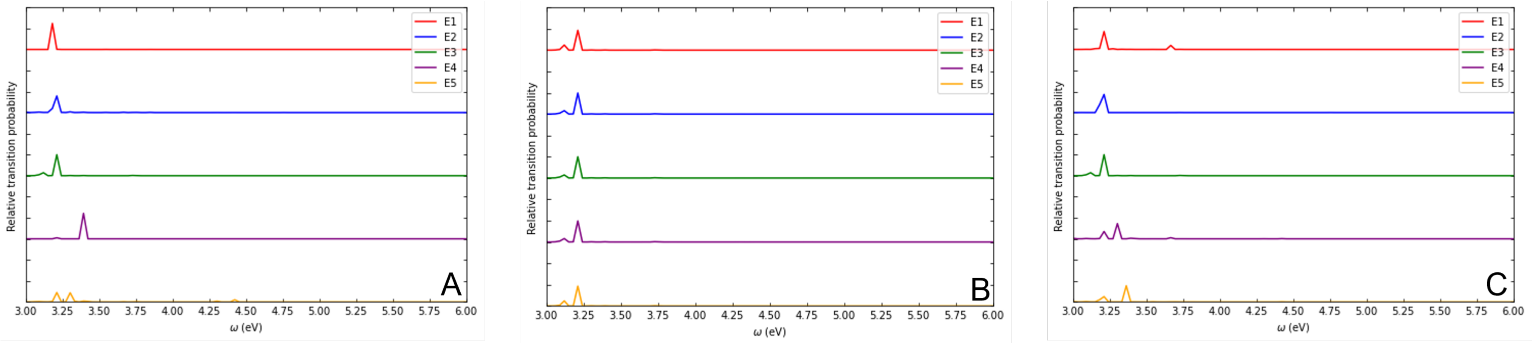


**Figure 12.** Here, we display plots of the relative transition probability (ordinate) versus $\omega$ (abscissa) for a $Pb_{52}Se_{52}$ quantum dot, in the presence of electric fields of differing strengths (E1, E2, E3, E4, and E5). Subplot A includes the spectra for when the electric fields are aligned with the quantum dot along the x-direction. Subplots B and C include the spectra for when the electric fields are aligned with the dot along the y-direction and z-direction, respectively.

## 4 Enhancing field-matter interaction

In figures 13-16, the IPE spectra (relative transition probability versus $\omega$) are displayed for the CdS, CdSe, PbS, and PbSe quantum dots when in the presence an electric field direction with which they are maximally-coupled. The spectra for when an external electric field is absent (E3) are also displayed in these plots. Again, it is important to note that the maximally-coupled electric field direction does not necessarily correspond to the field that results in the largest total relative transition probability. Instead, the field directions with which the dots are maximally-coupled produce the largest change in the maximum relative transition probabilities compared to maximum relative transition probabilities observed in the absence of an electric field.

For most of the quantum dots, the presence of the maximally-coupled electric field produces a noticeable shift in the energy of the emitted photon compared to energy of the emitted photon in the absence of an electric field, as is verified by Figure 13-Figure 16. For example, in subplot A of Figure 13 we see that when the $Cd_{24}S_{24}$ is in the presence of the maximally-couple field, the most probable energy of the emitted photon is 3.939 eV, while in the absence of an electric the most probable energy of the emitted photon is 3.091 eV. It is of paramount importance to note that these maximally-coupled electric fields are fields that result in either the greatest enhancement or de-enhancement of the inverse photoemission processes. For example, the maximally-coupled electric fields de-enhance the IPE processes in the $Pb_{44}S_{44}$ and $Pb_{140}S_{140}$ dots (Figure 15). Conversely, the maximally-coupled electric fields enhance the IPE processes in the CdS, CdSe, and PbSe QDs. In particular, especially strong enhancement of the IPE processes is observed for $Cd_{24}S_{24}$ (Figure 13) and $Pb_{29}Se_{29}$ (Figure 16).

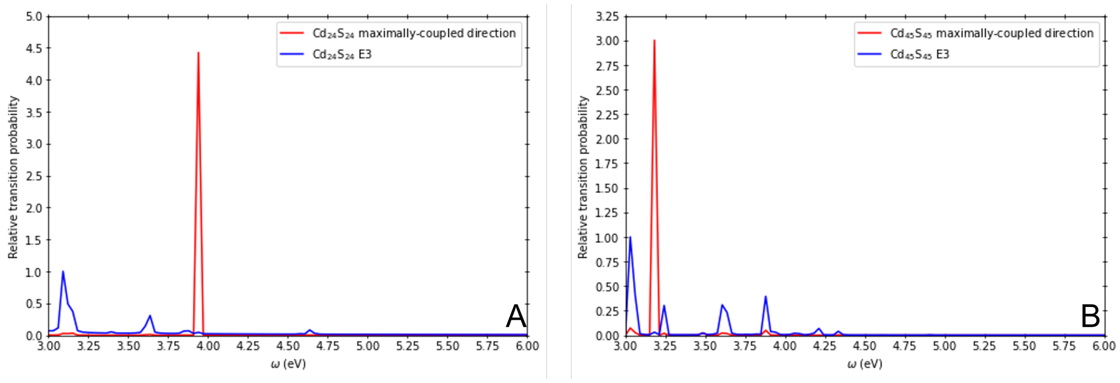


**Figure 13.** Subplots A and B (Relative transition probability versus $\omega$) contain the inverse photoemission spectra of $Cd_{24}S_{24}$ (subplot A) and $Cd_{45}S_{45}$ (subplot B), in the presence of electric fields with which the dots are maximally coupled, along with the spectra for the E3 field. For $Cd_{24}S_{24}$ the maximally-coupled electric field vector is $[1.0 \times 10^{-5}, -1.0 \times 10^{-5}, -1.0 \times 10^{-5}]$ and for $Cd_{45}S_{45}$ it is $[0.0, 1.0 \times 10^{-5}, 0.0]$.

We also see that when in the presence of the maximally-coupled fields the number of prominent peaks in the inverse PE spectra, for all of the systems studied, decreases compared to when an external electric field is absent. In subplot A of Figure 13, we see that there is one dominant peak, at 3.939 eV, of the spectrum for $Cd_{24}S_{24}$ in the presence of the maximally-coupled field, while there are multiple prominent peaks when an external field is absent. In subplot B of Figure 13, we also see that there is also only one dominant peak, at 3.182 eV, of the inverse PE spectrum for $Cd_{45}S_{45}$ in the presence of the maximally-coupled field, but multiple prominent peaks are observed when an external field is absent.

In subplot A of Figure 14, we see that there is one dominant peak, at 3.515 eV, of the spectrum for $Cd_{24}Se_{24}$ in the presence of the maximally-coupled field, while there are multiple prominent peaks when an external field is absent. In subplot B of Figure 14, again we see that there is only one dominant peak, at 4.000 eV, for the spectrum for $Cd_{54}Se_{54}$ in the presence of the maximally-coupled field, but again we see that multiple prominent peaks are observed when an external field is absent.

By examination of subplot A of Figure 15, we see that there are two prominent peaks of the spectrum for $Pb_{44}S_{44}$ in the presence of the maximally-coupled field. The most dominant peak for $Pb_{44}S_{44}$ in the presence of the maximally-coupled field and in the absence of an external field both occur at 3.091 eV. We also observe a peak at approximately 3.90 eV, on the spectrum for $Pb_{44}S_{44}$, in the presence of the maximally-coupled field. This particular peak is not present in the inverse PE spectrum of this dot when an external electric field is absent. In subplot B of Figure 15, we see that there are multiple prominent peaks on the spectrum $Pb_{140}S_{140}$ in the presence of the maximally-coupled field. The most prominent peak observed for this systems spectrum when the maximally-coupled field is present occurs at 3.455 eV. Additionally, by comparing subplot B of Figure 3 and subplot B of Figure 15 we observe that the peaks of the spectrum for $Pb_{140}S_{140}$ in the absence of an external field, occurring at approximately 3.05 eV and 3.65 eV, are greatly enhanced when the maximally-coupled field is present.

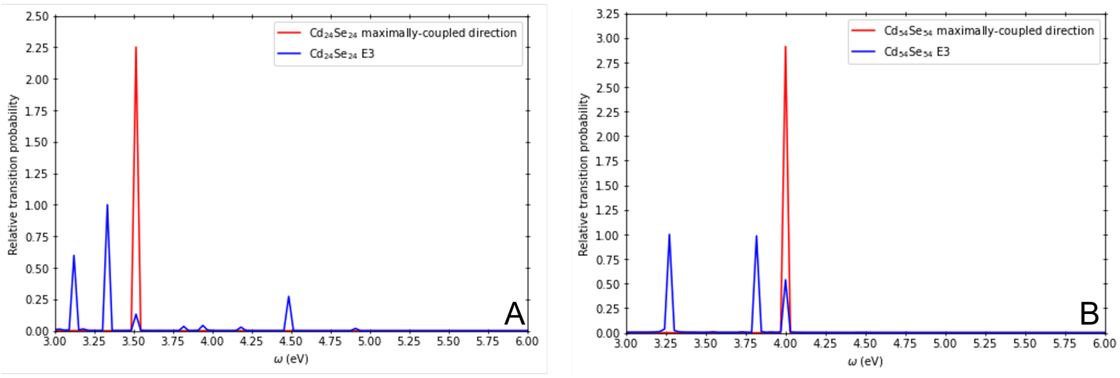


**Figure 14.** Subplots A and B (Relative transition probability versus $\omega$) contain the inverse photoemission spectra for $Cd_{24}Se_{24}$ (subplot A) and $Cd_{54}Se_{54}$ (subplot B), in the presence of electric fields with which the dots are maximally coupled, along with spectra for the E3 field. For $Cd_{24}Se_{24}$, the maximally-coupled electric field vector is $[0.00, -1.0 \times 10^{-5}, -1.0 \times 10^{-5}]$ and for $Cd_{54}Se_{54}$ it is $[-1.0 \times 10^{-5}, 0.00, -1.0 \times 10^{-5}]$.

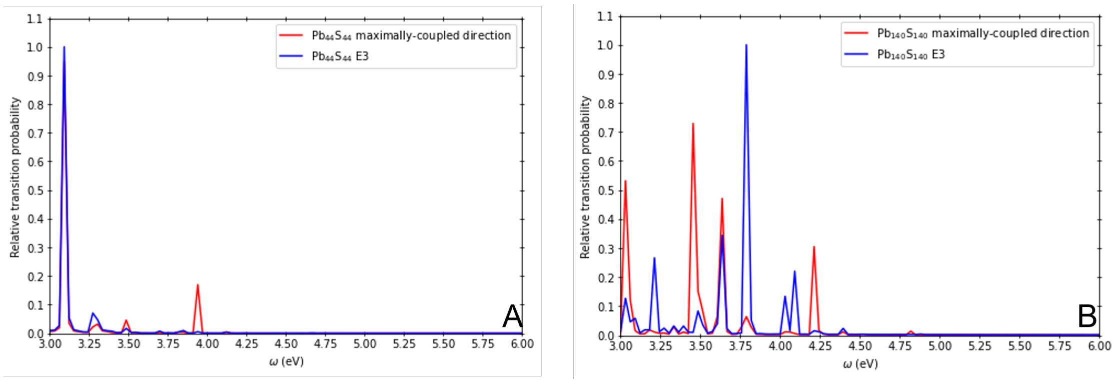


**Figure 15.** Subplots A and B (Relative transition probability versus $\omega$) contain the inverse photoemission spectra for $Pb_{44}S_{44}$ (subplot A) and $Pb_{140}S_{140}$ (subplot B), in the presence of electric fields with which the dots are maximally coupled, along with spectra for the E3 field. For $Pb_{44}S_{44}$, the maximally-coupled electric field vector is $[-1.0 \times 10^{-5}, -1.0 \times 10^{-5}, -1.0 \times 10^{-5}]$ and for $Pb_{140}S_{140}$ it is $[1.0 \times 10^{-5}, 1.0 \times 10^{-5}, -1.0 \times 10^{-5}]$.

In subplot A of Figure 16, we see that there is one dominant peak at 0.364 eV on the spectrum for $Pb_{29}Se_{29}$ in the presence of the maximally-coupled field, while there are multiple prominent peaks when an external field is absent. In subplot B of Figure 16, we also see that there is also only one dominant peak, at 3.394 eV, of the inverse PE spectrum for $Pb_{52}Se_{52}$ in the presence of the maximally-coupled field, while two prominent peaks are observed when an external field is absent. Additionally, we observe that for $Pb_{29}Se_{29}$ in the presence of the maximally-coupled field, the most probably energy of the emitted photon is 0.545 eV less than the most probable energy of the photon emitted when an external field is absent. When $Pb_{52}Se_{52}$ is in the presence of the maximally-coupled field, the most probably energy of the emitted photon is 0.182 eV greater than the most probable energy of the photon emitted when an external field is absent. When in the presence of the maximally-coupled fields, the number of prominent peaks in the inverse PE spectra of all of the systems studied, decreases compared to when an external electric field is absent.

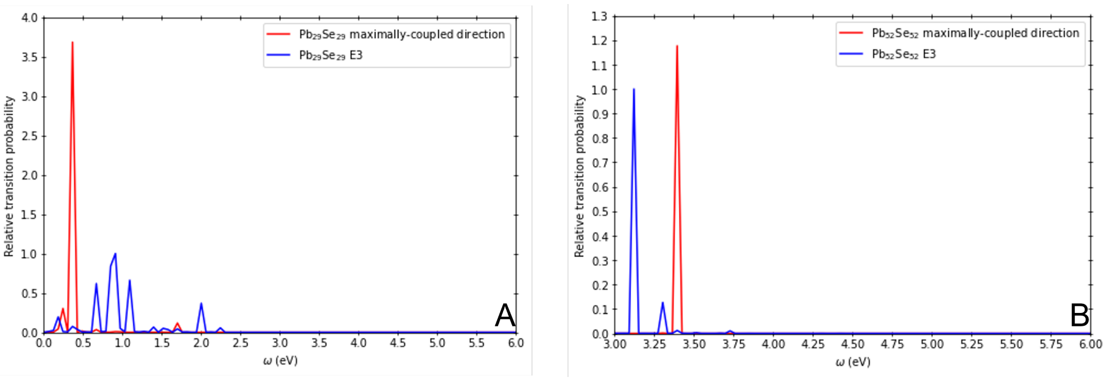


**Figure 16.** Subplots A and B (Relative transition probability versus $\omega$) contain the inverse photoemission spectra for $Pb_{29}Se_{29}$ (subplot A) and $Pb_{52}Se_{52}$ (subplot B), in the presence of electric fields with which the dots are maximally coupled, along with spectra for the E3 field. For $Pb_{29}Se_{29}$, the maximally-coupled electric field vector is $[-1.0\times10^{-5}, -1.0\times10^{-5}, 1.0\times10^{-5}]$ and for $Pb_{52}Se_{52}$ it is $[1.0\times10^{-5}, -1.0\times10^{-5}, 0.00]$.

## 5 List of Electric Vectors

**Table 1.** Electric Field Vector Information

| field | x-component | y-component | z-component |
|---|---|---|---|
| E1x | $-2.0 \times 10^{-5}$ | 0.0 | 0.0 |
| E2x | $-1.0 \times 10^{-5}$ | 0.0 | 0.0 |
| E3x | 0.0 | 0.0 | 0.0 |
| E4x | $1.0 \times 10^{-5}$ | 0.0 | 0.0 |
| E5x | $2.0 \times 10^{-5}$ | 0.0 | 0.0 |
| E1y | 0.00 | $-2.0 \times 10^{-5}$ | 0.0 |
| E2y | 0.00 | $-1.0 \times 10^{-5}$ | 0.0 |
| E3y | 0.0 | 0.0 | 0.0 |
| E4y | 0.00 | $1.0 \times 10^{-5}$ | 0.0 |
| E5y | 0.00 | $2.0 \times 10^{-5}$ | 0.0 |
| E1z | 0.00 | 0.00 | $-2.0 \times 10^{-5}$ |
| E2z | 0.00 | 0.00 | $-1.0 \times 10^{-5}$ |
| E3z | 0.0 | 0.0 | 0.0 |
| E4z | 0.00 | 0.00 | $1.0 \times 10^{-5}$ |
| E5z | 0.00 | 0.00 | $2.0 \times 10^{-5}$ |

## 6 Size of quantum dots

**Table 2.** Quantum dot diameter (nm)

| **Chemical System** | dot diameter |
|---|---|
| $Pb_{44}S_{44}$ | 1.542 |
| $Pb_{140}S_{140}$ | 2.280 |
| $Pb_{29}Se_{29}$ | 1.402 |
| $Pb_{52}Se_{52}$ | 1.590 |
| $Cd_{24}S_{24}$ | 1.266 |
| $Cd_{45}S_{45}$ | 1.504 |
| $Cd_{24}Se_{24}$ | 1.316 |
| $Cd_{54}Se_{54}$ | 1.792 |

## 7 HOMO-LUMO gap of quantum dots

**Table 3.** HOMO-LUMO gaps calculated from Hartree-Fock orbital energies

| **Chemical System** | HOMO-LUMO gap (eV) |
|---|---|
| $Pb_{44}S_{44}$ | 6.295 |
| $Pb_{140}S_{140}$ | 5.665 |
| $Pb_{29}Se_{29}$ | 3.018 |
| $Pb_{52}Se_{52}$ | 2.185 |
| $Cd_{24}S_{24}$ | 2.980 |
| $Cd_{45}S_{45}$ | 3.733 |
| $Cd_{24}Se_{24}$ | 2.754 |
| $Cd_{54}Se_{54}$ | 1.837 |

## 8 Stark-field induced mixing of molecular orbitals

The introduction of the Stark field causes the molecular orbitals (MO) of the field-free system to mix. In the absence of the field, the Fock operator $F^{(E=0)}$ is diagonal when expressed in terms of MOs. However, the presence of the field introduces a new interaction term, resulting in a field-dependent Fock operator (denoted $F^{(E)}$) that contains diagonal and non-diagonal elements. In block-matrix notation $F^{(E)}$ is given as,

$$\boldsymbol{F}^{(E)} = \begin{bmatrix} \boldsymbol{\varepsilon}_{\mathrm{o}} & \boldsymbol{0} \\ \boldsymbol{0} & \boldsymbol{\varepsilon}_{\mathrm{v}} \end{bmatrix} + \begin{bmatrix} \boldsymbol{V}_{\mathrm{oo}}^{(\mathrm{E})} & \boldsymbol{V}_{\mathrm{ov}}^{(\mathrm{E})} \\ \boldsymbol{V}_{\mathrm{vo}}^{(\mathrm{E})} & \boldsymbol{V}_{\mathrm{vv}}^{(\mathrm{E})} \end{bmatrix} \tag{26}$$

where, $o$ and $v$ represents occupied and virtual MOs. Diagonalization of the $F^{(E)}$ generate the new field-on orbital energies and wavefunction $\{\varepsilon_p^{(E)}, \chi_p^{(E)}\}$,

$$\boldsymbol{U}^{\dagger}\boldsymbol{F}^{(E)}\boldsymbol{U} = \begin{bmatrix} \boldsymbol{\varepsilon}_{\mathrm{o}}^{(E)} & \boldsymbol{0} \\ \boldsymbol{0} & \boldsymbol{\varepsilon}_{\mathrm{v}}^{(E)} \end{bmatrix} \tag{27}$$

where unitary matrix $\boldsymbol{U}$ diagonalizes the field-on Fock matrix. We note that $\boldsymbol{U}$ is field dependent

$$\boldsymbol{U}^{\dagger}\boldsymbol{U} = \boldsymbol{U}_E^{\dagger}\boldsymbol{U}_E = \boldsymbol{I} \tag{28}$$

and the column vectors of $U$ are the mixing coefficient for the field-on MOs.

$$\chi_p^{(E)} = \sum_{k=1}^{N_{\mathrm{o}}} U_{kp}\chi_k^{(E=0)} + \sum_{c=1}^{N_{\mathrm{v}}} U_{cp}\chi_c^{(E=0)} \tag{29}$$

The Stark field has a significant impact on the dipole-moment operator ($\mu$), which is responsible for the interaction between light and matter. In the presence of the Stark field, the matrix elements of the dipole-moment operator can be obtained by applying a unitary transformation to the field-off operators,

$$\boldsymbol{\mu}^{(E)} = \boldsymbol{U}_E^{\dagger} \begin{bmatrix} \boldsymbol{\mu}_{\text{oo}}^{(\text{E=0})} & \boldsymbol{\mu}_{\text{ov}}^{(\text{E=0})} \\ \boldsymbol{\mu}_{\text{vo}}^{(\text{E=0})} & \boldsymbol{\mu}_{\text{vv}}^{(\text{E=0})} \end{bmatrix} \boldsymbol{U}_E \tag{30}$$

Expanding the terms in term of occupied and virtual block matrices,

$$\boldsymbol{\mu}^{(E)} = \begin{bmatrix} \boldsymbol{U}_{\text{oo}}^{\dagger} & \boldsymbol{U}_{\text{ov}}^{\dagger} \\ \boldsymbol{U}_{\text{vo}}^{\dagger} & \boldsymbol{U}_{\text{vv}}^{\dagger} \end{bmatrix} \begin{bmatrix} \boldsymbol{\mu}_{\text{oo}}^{(\text{E=0})} & \boldsymbol{\mu}_{\text{ov}}^{(\text{E=0})} \\ \boldsymbol{\mu}_{\text{vo}}^{(\text{E=0})} & \boldsymbol{\mu}_{\text{vv}}^{(\text{E=0})} \end{bmatrix} \begin{bmatrix} \boldsymbol{U}_{\text{oo}} & \boldsymbol{U}_{\text{ov}} \\ \boldsymbol{U}_{\text{vo}} & \boldsymbol{U}_{\text{vv}} \end{bmatrix} \tag{31}$$

which gives the following expression,

$$\boldsymbol{\mu}^{(E)} = \begin{bmatrix} \boldsymbol{U}_{\text{oo}}^{\dagger}\boldsymbol{\mu}_{\text{oo}}^{(\text{E=0})}\boldsymbol{U}_{\text{oo}} + \boldsymbol{U}_{\text{oo}}^{\dagger}\boldsymbol{\mu}_{\text{ov}}^{(\text{E=0})}\boldsymbol{U}_{\text{vo}} + \boldsymbol{U}_{\text{ov}}^{\dagger}\boldsymbol{\mu}_{\text{vo}}^{(\text{E=0})}\boldsymbol{U}_{\text{oo}} + \boldsymbol{U}_{\text{ov}}^{\dagger}\boldsymbol{\mu}_{\text{vv}}^{(\text{E=0})}\boldsymbol{U}_{\text{vo}} & \boldsymbol{U}_{\text{oo}}^{\dagger}\boldsymbol{\mu}_{\text{oo}}^{(\text{E=0})}\boldsymbol{U}_{\text{ov}} + \boldsymbol{U}_{\text{oo}}^{\dagger}\boldsymbol{\mu}_{\text{ov}}^{(\text{E=0})}\boldsymbol{U}_{\text{vv}} + \boldsymbol{U}_{\text{ov}}^{\dagger}\boldsymbol{\mu}_{\text{vo}}^{(\text{E=0})}\boldsymbol{U}_{\text{ov}} + \boldsymbol{U}_{\text{ov}}^{\dagger}\boldsymbol{\mu}_{\text{vv}}^{(\text{E=0})}\boldsymbol{U}_{\text{vv}} \\ \boldsymbol{U}_{\text{vo}}^{\dagger}\boldsymbol{\mu}_{\text{oo}}^{(\text{E=0})}\boldsymbol{U}_{\text{oo}} + \boldsymbol{U}_{\text{vo}}^{\dagger}\boldsymbol{\mu}_{\text{ov}}^{(\text{E=0})}\boldsymbol{U}_{\text{vo}} + \boldsymbol{U}_{\text{vv}}^{\dagger}\boldsymbol{\mu}_{\text{vo}}^{(\text{E=0})}\boldsymbol{U}_{\text{oo}} + \boldsymbol{U}_{\text{vv}}^{\dagger}\boldsymbol{\mu}_{\text{vv}}^{(\text{E=0})}\boldsymbol{U}_{\text{vo}} & \boldsymbol{U}_{\text{vo}}^{\dagger}\boldsymbol{\mu}_{\text{oo}}^{(\text{E=0})}\boldsymbol{U}_{\text{ov}} + \boldsymbol{U}_{\text{vo}}^{\dagger}\boldsymbol{\mu}_{\text{ov}}^{(\text{E=0})}\boldsymbol{U}_{\text{vv}} + \boldsymbol{U}_{\text{vv}}^{\dagger}\boldsymbol{\mu}_{\text{vo}}^{(\text{E=0})}\boldsymbol{U}_{\text{ov}} + \boldsymbol{U}_{\text{vv}}^{\dagger}\boldsymbol{\mu}_{\text{vv}}^{(\text{E=0})}\boldsymbol{U}_{\text{vv}} \end{bmatrix} \tag{32}$$

Analysis of the field-on virtual-virtual block of $\boldsymbol{\mu}$ which is responsible for intra-band transitions

$$\boldsymbol{\mu}_{\text{vv}}^{(E)} = \boldsymbol{U}_{\text{vv}}^{\dagger}\boldsymbol{\mu}_{\text{vv}}^{(\text{E=0})}\boldsymbol{U}_{\text{vv}} + \boldsymbol{U}_{\text{vo}}^{\dagger}\boldsymbol{\mu}_{\text{oo}}^{(\text{E=0})}\boldsymbol{U}_{\text{ov}} + \boldsymbol{U}_{\text{vo}}^{\dagger}\boldsymbol{\mu}_{\text{ov}}^{(\text{E=0})}\boldsymbol{U}_{\text{vv}} + \boldsymbol{U}_{\text{vv}}^{\dagger}\boldsymbol{\mu}_{\text{vo}}^{(\text{E=0})}\boldsymbol{U}_{\text{ov}} \tag{33}$$

reveal that the field-on $\boldsymbol{\mu}_{\text{vv}}^{(E)}$ block not only depends on the field-off virtual-virtual block, but also on field-off occupied-occupied and occupied-virtual blocks.

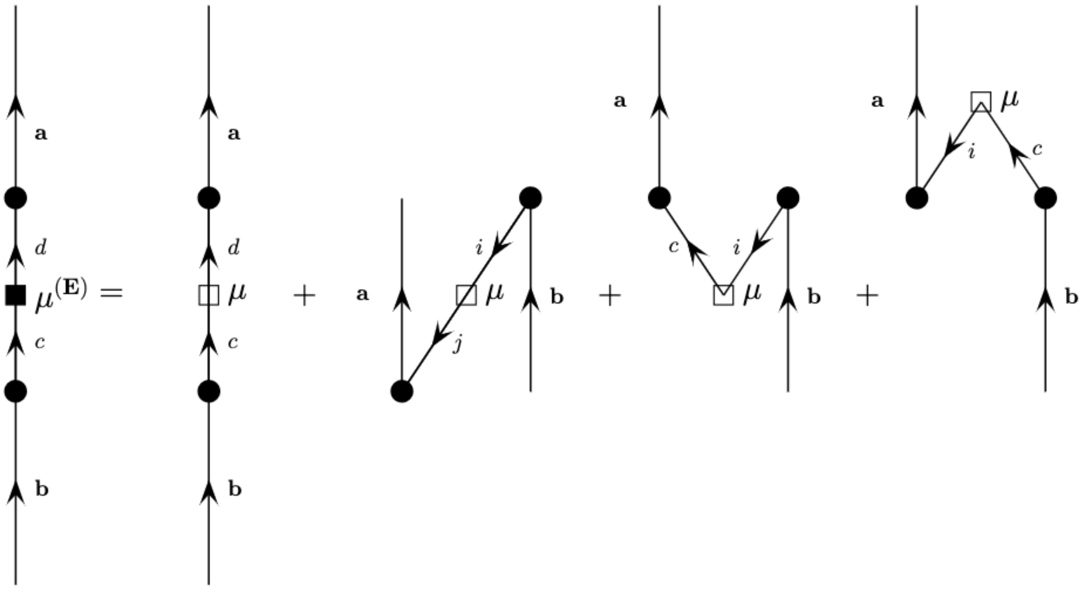


**Figure 17.** Feynman-Goldstone diagrams of the contributing terms to the field-dependent dipole-moment operator. The filled boxes and circles represent field-dependent quantities, while the unfilled boxes represent field-independent quantities.

## 9 Cartesian coordinates of $Cd_{24}S_{24}$ in atomic units

```
atom      x              y                z
S -3.90791426 -11.28131214 -0.79285816
Cd -7.81594577 -9.02500910 0.79285816
Cd -7.81586761 -4.51253839 -5.55000712
S -7.81586761 -4.51253839 -0.79285816
Cd -11.72389911 -2.25623535 0.79285816
Cd -7.81586761 -4.51253839 7.13572344
S -11.72382095 2.25623535 -0.79285816
S -0.00003908 -9.02500910 -7.13572344
S 3.90799242 -11.28131214 -0.79285816
Cd -0.00003908 -9.02500910 0.79285816
S -0.00003908 -9.02500910 5.55000712
Cd 0.00003908 -4.51253839 -5.55000712
S -3.90799242 -2.25623535 -7.13572344
S 0.00003908 -4.51253839 -0.79285816
Cd -3.90799242 -2.25623535 0.79285816
Cd 0.00003908 -4.51253839 7.13572344
S -3.90799242 -2.25623535 5.55000712
Cd -3.90791426 2.25623535 -5.55000712
S -7.81594577 4.51253839 -7.13572344
S -3.90791426 2.25623535 -0.79285816
Cd -7.81594577 4.51253839 0.79285816
Cd -3.90791426 2.25623535 7.13572344
S -7.81594577 4.51253839 5.55000712
S -7.81586761 9.02500910 -0.79285816
Cd 7.81586761 -9.02500910 0.79285816
Cd 7.81594577 -4.51253839 -5.55000712
S 3.90791426 -2.25623535 -7.13572344
S 7.81594577 -4.51253839 -0.79285816
Cd 3.90791426 -2.25623535 0.79285816
Cd 7.81594577 -4.51253839 7.13572344
S 3.90791426 -2.25623535 5.55000712
Cd 3.90799242 2.25623535 -5.55000712
S -0.00003908 4.51253839 -7.13572344
S 3.90799242 2.25623535 -0.79285816
Cd -0.00003908 4.51253839 0.79285816
Cd 3.90799242 2.25623535 7.13572344
S -0.00003908 4.51253839 5.55000712
Cd 0.00003908 9.02500910 -5.55000712
S 0.00003908 9.02500910 -0.79285816
Cd -3.90799242 11.28131214 0.79285816
Cd 0.00003908 9.02500910 7.13572344
Cd 11.72382095 -2.25623535 0.79285816
S 7.81586761 4.51253839 -7.13572344
S 11.72389911 2.25623535 -0.79285816
Cd 7.81586761 4.51253839 0.79285816
S 7.81586761 4.51253839 5.55000712
S 7.81594577 9.02500910 -0.79285816
Cd 3.90791426 11.28131214 0.79285816
```

## 10 Cartesian coordinates of $Cd_{45}S_{45}$ in atomic units

```
atom      x              y               z
Cd -3.90791426 -11.28131214 -5.55000712
S -7.81594577 -9.02500910 -7.13572344
S -3.90791426 -11.28131214 -0.79285816
Cd -7.81594577 -9.02500910 0.79285816
Cd -3.90791426 -11.28131214 7.13572344
S -7.81594577 -9.02500910 5.55000712
Cd -7.81586761 -4.51253839 -5.55000712
S -11.72389911 -2.25623535 -7.13572344
S -7.81586761 -4.51253839 -0.79285816
Cd -11.72389911 -2.25623535 0.79285816
Cd -7.81586761 -4.51253839 7.13572344
S -11.72389911 -2.25623535 5.55000712
S -7.81586761 -4.51253839 11.89287240
Cd -11.72382095 2.25623535 -5.55000712
S -11.72382095 2.25623535 -0.79285816
Cd -11.72382095 2.25623535 7.13572344
Cd -0.00003908 -9.02500910 -11.89287240
Cd 3.90799242 -11.28131214 -5.55000712
S -0.00003908 -9.02500910 -7.13572344
S 3.90799242 -11.28131214 -0.79285816
Cd -0.00003908 -9.02500910 0.79285816
Cd 3.90799242 -11.28131214 7.13572344
S -0.00003908 -9.02500910 5.55000712
S 0.00003908 -4.51253839 -13.47858872
Cd -3.90799242 -2.25623535 -11.89287240
Cd 0.00003908 -4.51253839 -5.55000712
S -3.90799242 -2.25623535 -7.13572344
S 0.00003908 -4.51253839 -0.79285816
Cd -3.90799242 -2.25623535 0.79285816
Cd 0.00003908 -4.51253839 7.13572344
S -3.90799242 -2.25623535 5.55000712
S 0.00003908 -4.51253839 11.89287240
Cd -3.90799242 -2.25623535 13.47858872
S -3.90791426 2.25623535 -13.47858872
Cd -7.81594577 4.51253839 -11.89287240
Cd -3.90791426 2.25623535 -5.55000712
S -7.81594577 4.51253839 -7.13572344
S -3.90791426 2.25623535 -0.79285816
Cd -7.81594577 4.51253839 0.79285816
Cd -3.90791426 2.25623535 7.13572344
S -7.81594577 4.51253839 5.55000712
S -3.90791426 2.25623535 11.89287240
Cd -7.81586761 9.02500910 -5.55000712
S -7.81586761 9.02500910 -0.79285816
Cd -7.81586761 9.02500910 7.13572344
S 7.81586761 -9.02500910 -7.13572344
Cd 7.81586761 -9.02500910 0.79285816
S 7.81586761 -9.02500910 5.55000712
Cd 3.90791426 -2.25623535 -11.89287240
Cd 7.81594577 -4.51253839 -5.55000712
S 3.90791426 -2.25623535 -7.13572344
S 7.81594577 -4.51253839 -0.79285816
Cd 3.90791426 -2.25623535 0.79285816
```

Cd 7.81594577 -4.51253839 7.13572344
S 3.90791426 -2.25623535 5.55000712
S 7.81594577 -4.51253839 11.89287240
Cd 3.90791426 -2.25623535 13.47858872
S 3.90799242 2.25623535 -13.47858872
Cd -0.00003908 4.51253839 -11.89287240
Cd 3.90799242 2.25623535 -5.55000712
S -0.00003908 4.51253839 -7.13572344
S 3.90799242 2.25623535 -0.79285816
Cd -0.00003908 4.51253839 0.79285816
Cd 3.90799242 2.25623535 7.13572344
S -0.00003908 4.51253839 5.55000712
S 3.90799242 2.25623535 11.89287240
Cd -0.00003908 4.51253839 13.47858872
Cd 0.00003908 9.02500910 -5.55000712
S -3.90799242 11.28131214 -7.13572344
S 0.00003908 9.02500910 -0.79285816
Cd -3.90799242 11.28131214 0.79285816
Cd 0.00003908 9.02500910 7.13572344
S -3.90799242 11.28131214 5.55000712
S 0.00003908 9.02500910 11.89287240
S 11.72382095 -2.25623535 -7.13572344
Cd 11.72382095 -2.25623535 0.79285816
S 11.72382095 -2.25623535 5.55000712
Cd 7.81586761 4.51253839 -11.89287240
Cd 11.72389911 2.25623535 -5.55000712
S 7.81586761 4.51253839 -7.13572344
S 11.72389911 2.25623535 -0.79285816
Cd 7.81586761 4.51253839 0.79285816
Cd 11.72389911 2.25623535 7.13572344
S 7.81586761 4.51253839 5.55000712
Cd 7.81594577 9.02500910 -5.55000712
S 3.90791426 11.28131214 -7.13572344
S 7.81594577 9.02500910 -0.79285816
Cd 3.90791426 11.28131214 0.79285816
Cd 7.81594577 9.02500910 7.13572344
S 3.90791426 11.28131214 5.55000712

## 11 Cartesian coordinates of $Cd_{24}Se_{24}$ in atomic units

```
atom      x              y               z
Se -8.12339331 -9.37938316 -0.82855036
Cd -4.06108743 -11.72475655 0.82855036
Se -8.12258101 -4.69004331 -7.45695324
Se -12.18488689 -2.34466992 -0.82855036
Cd -8.12258101 -4.69004331 0.82855036
Se -8.12258101 -4.69004331 5.79985252
Cd -12.18407459 2.34466992 0.82855036
Cd -0.00040615 -9.37938316 -5.79985252
Se -0.00040615 -9.37938316 -0.82855036
Cd 4.06189973 -11.72475655 0.82855036
Cd -0.00040615 -9.37938316 7.45695324
Cd -4.06189973 -2.34466992 -5.79985252
Se 0.00040615 -4.69004331 -7.45695324
Se -4.06189973 -2.34466992 -0.82855036
Cd 0.00040615 -4.69004331 0.82855036
Cd -4.06189973 -2.34466992 7.45695324
Se 0.00040615 -4.69004331 5.79985252
Cd -8.12339331 4.69004332 -5.79985252
Se -4.06108743 2.34466992 -7.45695324
Se -8.12339331 4.69004332 -0.82855036
Cd -4.06108743 2.34466992 0.82855036
Cd -8.12339331 4.69004332 7.45695324
Se -4.06108743 2.34466992 5.79985252
Cd -8.12258101 9.37938316 0.82855036
Se 8.12258101 -9.37938316 -0.82855036
Cd 4.06108743 -2.34466992 -5.79985252
Se 8.12339331 -4.69004331 -7.45695324
Se 4.06108743 -2.34466992 -0.82855036
Cd 8.12339331 -4.69004331 0.82855036
Cd 4.06108743 -2.34466992 7.45695324
Se 8.12339331 -4.69004331 5.79985252
Cd -0.00040615 4.69004332 -5.79985252
Se 4.06189973 2.34466992 -7.45695324
Se -0.00040615 4.69004332 -0.82855036
Cd 4.06189973 2.34466992 0.82855036
Cd -0.00040615 4.69004332 7.45695324
Se 4.06189973 2.34466992 5.79985252
Se 0.00040615 9.37938316 -7.45695324
Se -4.06189973 11.72475655 -0.82855036
Cd 0.00040615 9.37938316 0.82855036
Se 0.00040615 9.37938316 5.79985252
Se 12.18407459 -2.34466992 -0.82855036
Cd 8.12258101 4.69004332 -5.79985252
Se 8.12258101 4.69004332 -0.82855036
Cd 12.18488689 2.34466992 0.82855036
Cd 8.12258101 4.69004332 7.45695324
Se 4.06108743 11.72475655 -0.82855036
Cd 8.12339331 9.37938316 0.82855036
```

## 12 Cartesian coordinates of $Cd_{54}Se_{54}$ in atomic units

| atom | x | y | z |
|---|---|---|---|
| Cd | -12.18407459 | -11.72475655 | 0.82855036 |
| Cd | -16.24556817 | -4.69004331 | 0.82855036 |
| Se | -4.06189973 | -16.41409640 | -0.82855036 |
| Cd | -8.12339331 | -9.37938316 | -5.79985252 |
| Se | -4.06108743 | -11.72475655 | -7.45695324 |
| Se | -8.12339331 | -9.37938316 | -0.82855036 |
| Cd | -4.06108743 | -11.72475655 | 0.82855036 |
| Cd | -8.12339331 | -9.37938316 | 7.45695324 |
| Se | -4.06108743 | -11.72475655 | 5.79985252 |
| Cd | -8.12258101 | -4.69004331 | -12.42825540 |
| Cd | -12.18488689 | -2.34466992 | -5.79985252 |
| Se | -8.12258101 | -4.69004331 | -7.45695324 |
| Se | -12.18488689 | -2.34466992 | -0.82855036 |
| Cd | -8.12258101 | -4.69004331 | 0.82855036 |
| Cd | -12.18488689 | -2.34466992 | 7.45695324 |
| Se | -8.12258101 | -4.69004331 | 5.79985252 |
| Cd | -8.12258101 | -4.69004331 | 14.08535611 |
| Se | -12.18407459 | 2.34466992 | -7.45695324 |
| Se | -16.24638047 | 4.69004332 | -0.82855036 |
| Cd | -12.18407459 | 2.34466992 | 0.82855036 |
| Se | -12.18407459 | 2.34466992 | 5.79985252 |
| Se | 4.06108743 | -16.41409640 | -0.82855036 |
| Se | -0.00040615 | -9.37938316 | -14.08535611 |
| Cd | -0.00040615 | -9.37938316 | -5.79985252 |
| Se | 4.06189973 | -11.72475655 | -7.45695324 |
| Se | -0.00040615 | -9.37938316 | -0.82855036 |
| Cd | 4.06189973 | -11.72475655 | 0.82855036 |
| Cd | -0.00040615 | -9.37938316 | 7.45695324 |
| Se | 4.06189973 | -11.72475655 | 5.79985252 |
| Se | -0.00040615 | -9.37938316 | 12.42825540 |
| Se | -4.06189973 | -2.34466992 | -14.08535611 |
| Cd | 0.00040615 | -4.69004331 | -12.42825540 |
| Cd | -4.06189973 | -2.34466992 | -5.79985252 |
| Se | 0.00040615 | -4.69004331 | -7.45695324 |
| Se | -4.06189973 | -2.34466992 | -0.82855036 |
| Cd | 0.00040615 | -4.69004331 | 0.82855036 |
| Cd | -4.06189973 | -2.34466992 | 7.45695324 |
| Se | 0.00040615 | -4.69004331 | 5.79985252 |
| Se | -4.06189973 | -2.34466992 | 12.42825540 |
| Cd | 0.00040615 | -4.69004331 | 14.08535611 |
| Se | -8.12339331 | 4.69004332 | -14.08535611 |
| Cd | -4.06108743 | 2.34466992 | -12.42825540 |
| Cd | -8.12339331 | 4.69004332 | -5.79985252 |
| Se | -4.06108743 | 2.34466992 | -7.45695324 |
| Se | -8.12339331 | 4.69004332 | -0.82855036 |
| Cd | -4.06108743 | 2.34466992 | 0.82855036 |
| Cd | -8.12339331 | 4.69004332 | 7.45695324 |
| Se | -4.06108743 | 2.34466992 | 5.79985252 |
| Se | -8.12339331 | 4.69004332 | 12.42825540 |
| Cd | -4.06108743 | 2.34466992 | 14.08535611 |
| Se | -8.12258101 | 9.37938316 | -7.45695324 |
| Se | -12.18488689 | 11.72475655 | -0.82855036 |
| Cd | -8.12258101 | 9.37938316 | 0.82855036 |

Se -8.12258101 9.37938316 5.79985252
Cd 8.12258101 -9.37938316 -5.79985252
Se 8.12258101 -9.37938316 -0.82855036
Cd 12.18488689 -11.72475655 0.82855036
Cd 8.12258101 -9.37938316 7.45695324
Se 4.06108743 -2.34466992 -14.08535611
Cd 8.12339331 -4.69004331 -12.42825540
Cd 4.06108743 -2.34466992 -5.79985252
Se 8.12339331 -4.69004331 -7.45695324
Se 4.06108743 -2.34466992 -0.82855036
Cd 8.12339331 -4.69004331 0.82855036
Cd 4.06108743 -2.34466992 7.45695324
Se 8.12339331 -4.69004331 5.79985252
Se 4.06108743 -2.34466992 12.42825540
Cd 8.12339331 -4.69004331 14.08535611
Se -0.00040615 4.69004332 -14.08535611
Cd 4.06189973 2.34466992 -12.42825540
Cd -0.00040615 4.69004332 -5.79985252
Se 4.06189973 2.34466992 -7.45695324
Se -0.00040615 4.69004332 -0.82855036
Cd 4.06189973 2.34466992 0.82855036
Cd -0.00040615 4.69004332 7.45695324
Se 4.06189973 2.34466992 5.79985252
Se -0.00040615 4.69004332 12.42825540
Cd 4.06189973 2.34466992 14.08535611
Cd 0.00040615 9.37938316 -12.42825540
Cd -4.06189973 11.72475655 -5.79985252
Se 0.00040615 9.37938316 -7.45695324
Se -4.06189973 11.72475655 -0.82855036
Cd 0.00040615 9.37938316 0.82855036
Cd -4.06189973 11.72475655 7.45695324
Se 0.00040615 9.37938316 5.79985252
Cd 0.00040615 9.37938316 14.08535611
Cd -4.06108743 16.41409640 0.82855036
Cd 12.18407459 -2.34466992 -5.79985252
Se 12.18407459 -2.34466992 -0.82855036
Cd 16.24638047 -4.69004331 0.82855036
Cd 12.18407459 -2.34466992 7.45695324
Se 8.12258101 4.69004332 -14.08535611
Cd 8.12258101 4.69004332 -5.79985252
Se 12.18488689 2.34466992 -7.45695324
Se 8.12258101 4.69004332 -0.82855036
Cd 12.18488689 2.34466992 0.82855036
Cd 8.12258101 4.69004332 7.45695324
Se 12.18488689 2.34466992 5.79985252
Se 8.12258101 4.69004332 12.42825540
Cd 4.06108743 11.72475655 -5.79985252
Se 8.12339331 9.37938316 -7.45695324
Se 4.06108743 11.72475655 -0.82855036
Cd 8.12339331 9.37938316 0.82855036
Cd 4.06108743 11.72475655 7.45695324
Se 8.12339331 9.37938316 5.79985252
Cd 4.06189973 16.41409640 0.82855036
Se 16.24556817 4.69004332 -0.82855036
Se 12.18407459 11.72475655 -0.82855036

## 13 Cartesian coordinates of $Pb_{29}Se_{29}$ in atomic units

```
atom      x              y               z
Se -13.01076343 0.00000000 -2.89128076
Pb -7.22820191 -11.56512305 -2.89128076
Se -1.44564038 -11.56512305 -2.89128076
Pb -1.44564038 -5.78256153 -2.89128076
Pb -7.22820191 0.00000000 -2.89128076
Pb -7.22820191 -5.78256153 -8.67384229
Se -1.44564038 -5.78256153 -8.67384229
Se -1.44564038 0.00000000 -2.89128076
Se -7.22820191 -5.78256153 -2.89128076
Pb -1.44564038 -5.78256153 8.67384229
Pb -7.22820191 0.00000000 8.67384229
Pb -7.22820191 -5.78256153 2.89128076
Se -1.44564038 -5.78256153 2.89128076
Se -1.44564038 0.00000000 8.67384229
Se -7.22820191 -11.56512305 2.89128076
Se -7.22820191 -5.78256153 8.67384229
Pb -1.44564038 5.78256153 -2.89128076
Pb -7.22820191 11.56512305 -2.89128076
Pb -7.22820191 5.78256153 -8.67384229
Se -1.44564038 5.78256153 -8.67384229
Se -1.44564038 11.56512305 -2.89128076
Se -7.22820191 0.00000000 -8.67384229
Se -7.22820191 5.78256153 -2.89128076
Pb -13.01076343 0.00000000 2.89128076
Pb -1.44564038 5.78256153 8.67384229
Pb -7.22820191 5.78256153 2.89128076
Se -1.44564038 5.78256153 2.89128076
Se -7.22820191 0.00000000 2.89128076
Se -7.22820191 5.78256153 8.67384229
Se -7.22820191 11.56512305 2.89128076
Pb 4.33692114 -11.56512305 -2.89128076
Pb 10.11948267 -5.78256153 -2.89128076
Pb 4.33692114 0.00000000 -2.89128076
Pb 4.33692114 -5.78256153 -8.67384229
Se 10.11948267 0.00000000 -2.89128076
Se 4.33692114 -5.78256153 -2.89128076
Pb -1.44564038 -11.56512305 2.89128076
Pb 4.33692114 0.00000000 8.67384229
Pb 4.33692114 -5.78256153 2.89128076
Se 10.11948267 -5.78256153 2.89128076
Se 10.11948267 0.00000000 8.67384229
Se 4.33692114 -11.56512305 2.89128076
Se 4.33692114 -5.78256153 8.67384229
Pb -1.44564038 0.00000000 -8.67384229
Pb 10.11948267 5.78256153 -2.89128076
Pb 4.33692114 11.56512305 -2.89128076
Pb 4.33692114 5.78256153 -8.67384229
Se 4.33692114 0.00000000 -8.67384229
Se 4.33692114 5.78256153 -2.89128076
Pb -1.44564038 0.00000000 2.89128076
Pb 4.33692114 5.78256153 2.89128076
Se 10.11948267 5.78256153 2.89128076
Se 4.33692114 0.00000000 2.89128076
```

```
Se 4.33692114 5.78256153 8.67384229
Pb -1.44564038 11.56512305 2.89128076
Se 4.33692114 11.56512305 2.89128076
Pb 10.11948267 0.00000000 -8.67384229
Pb 10.11948267 0.00000000 2.89128076
```

## 14 Cartesian coordinates of $Pb_{52}Se_{52}$ in atomic units

```
atom      x              y                z
Pb -13.01076343 -5.78256153 -2.89128076
Se -13.01076343 -5.78256153 -8.67384229
Se -13.01076343 0.00000000 -2.89128076
Pb -13.01076343 -5.78256153 8.67384229
Se -13.01076343 -5.78256153 2.89128076
Se -13.01076343 0.00000000 8.67384229
Pb -13.01076343 5.78256153 -2.89128076
Se -13.01076343 5.78256153 -8.67384229
Pb -13.01076343 5.78256153 8.67384229
Se -13.01076343 5.78256153 2.89128076
Pb -7.22820191 -11.56512305 -2.89128076
Se -1.44564038 -11.56512305 -2.89128076
Pb -7.22820191 -11.56512305 8.67384229
Se -1.44564038 -11.56512305 8.67384229
Pb -1.44564038 -5.78256153 -14.45640381
Pb -7.22820191 0.00000000 -14.45640381
Se -1.44564038 0.00000000 -14.45640381
Pb -1.44564038 -5.78256153 -2.89128076
Pb -7.22820191 0.00000000 -2.89128076
Pb -7.22820191 -5.78256153 -8.67384229
Se -1.44564038 -5.78256153 -8.67384229
Se -1.44564038 0.00000000 -2.89128076
Se -7.22820191 -11.56512305 -8.67384229
Se -7.22820191 -5.78256153 -2.89128076
Pb -1.44564038 -5.78256153 8.67384229
Pb -7.22820191 0.00000000 8.67384229
Pb -7.22820191 -5.78256153 2.89128076
Se -1.44564038 -5.78256153 2.89128076
Se -1.44564038 0.00000000 8.67384229
Se -7.22820191 -11.56512305 2.89128076
Se -7.22820191 -5.78256153 8.67384229
Se -1.44564038 -5.78256153 14.45640381
Pb -1.44564038 5.78256153 -14.45640381
Pb -13.01076343 0.00000000 -8.67384229
Pb -1.44564038 5.78256153 -2.89128076
Pb -7.22820191 11.56512305 -2.89128076
Pb -7.22820191 5.78256153 -8.67384229
Se -1.44564038 5.78256153 -8.67384229
Se -1.44564038 11.56512305 -2.89128076
Se -7.22820191 0.00000000 -8.67384229
Se -7.22820191 5.78256153 -2.89128076
Pb -13.01076343 0.00000000 2.89128076
Pb -1.44564038 5.78256153 8.67384229
Pb -7.22820191 11.56512305 8.67384229
Pb -7.22820191 5.78256153 2.89128076
Se -1.44564038 5.78256153 2.89128076
Se -1.44564038 11.56512305 8.67384229
Se -7.22820191 0.00000000 2.89128076
Se -7.22820191 5.78256153 8.67384229
Se -1.44564038 5.78256153 14.45640381
Se -7.22820191 0.00000000 14.45640381
Se -7.22820191 11.56512305 -8.67384229
Se -7.22820191 11.56512305 2.89128076
```

Pb 4.33692114 -11.56512305 -2.89128076
Se 10.11948267 -11.56512305 -2.89128076
Pb 4.33692114 -11.56512305 8.67384229
Pb 4.33692114 0.00000000 -14.45640381
Se 4.33692114 -5.78256153 -14.45640381
Pb -1.44564038 -11.56512305 -8.67384229
Pb 10.11948267 -5.78256153 -2.89128076
Pb 4.33692114 0.00000000 -2.89128076
Pb 4.33692114 -5.78256153 -8.67384229
Se 10.11948267 -5.78256153 -8.67384229
Se 10.11948267 0.00000000 -2.89128076
Se 4.33692114 -11.56512305 -8.67384229
Se 4.33692114 -5.78256153 -2.89128076
Pb -1.44564038 -11.56512305 2.89128076
Pb 10.11948267 -5.78256153 8.67384229
Pb 4.33692114 0.00000000 8.67384229
Pb 4.33692114 -5.78256153 2.89128076
Se 10.11948267 -5.78256153 2.89128076
Se 10.11948267 0.00000000 8.67384229
Se 4.33692114 -11.56512305 2.89128076
Se 4.33692114 -5.78256153 8.67384229
Pb 4.33692114 -5.78256153 14.45640381
Se 4.33692114 5.78256153 -14.45640381
Pb -1.44564038 0.00000000 -8.67384229
Pb 10.11948267 5.78256153 -2.89128076
Pb 4.33692114 11.56512305 -2.89128076
Pb 4.33692114 5.78256153 -8.67384229
Se 10.11948267 5.78256153 -8.67384229
Se 10.11948267 11.56512305 -2.89128076
Se 4.33692114 0.00000000 -8.67384229
Se 4.33692114 5.78256153 -2.89128076
Pb -1.44564038 0.00000000 2.89128076
Pb 10.11948267 5.78256153 8.67384229
Pb 4.33692114 11.56512305 8.67384229
Pb 4.33692114 5.78256153 2.89128076
Se 10.11948267 5.78256153 2.89128076
Se 4.33692114 0.00000000 2.89128076
Se 4.33692114 5.78256153 8.67384229
Pb -1.44564038 0.00000000 14.45640381
Pb 4.33692114 5.78256153 14.45640381
Se 4.33692114 0.00000000 14.45640381
Pb -1.44564038 11.56512305 -8.67384229
Se 4.33692114 11.56512305 -8.67384229
Pb -1.44564038 11.56512305 2.89128076
Se 4.33692114 11.56512305 2.89128076
Pb 15.90204419 0.00000000 -2.89128076
Pb 10.11948267 -11.56512305 2.89128076
Pb 10.11948267 0.00000000 -8.67384229
Pb 10.11948267 0.00000000 2.89128076
Se 15.90204419 0.00000000 2.89128076
Pb 10.11948267 11.56512305 2.89128076

## 15 Cartesian coordinates of $Pb_{44}S_{44}$ in atomic units

```
atom      x              y                z
Pb 2.80435337 2.80435337 -14.02176684
S -2.80435337 2.80435337 -14.02176684
S 2.80435337 -2.80435337 -14.02176684
Pb -2.80435337 -2.80435337 -14.02176684
S 8.41306010 8.41306010 -8.41306010
Pb 2.80435337 8.41306010 -8.41306010
Pb 8.41306010 2.80435337 -8.41306010
S 2.80435337 2.80435337 -8.41306010
Pb -8.41306010 8.41306010 -8.41306010
Pb -2.80435337 2.80435337 -8.41306010
S -2.80435337 8.41306010 -8.41306010
S -8.41306010 2.80435337 -8.41306010
Pb 2.80435337 -2.80435337 -8.41306010
Pb 8.41306010 -8.41306010 -8.41306010
S 8.41306010 -2.80435337 -8.41306010
S 2.80435337 -8.41306010 -8.41306010
Pb -8.41306010 -2.80435337 -8.41306010
Pb -2.80435337 -8.41306010 -8.41306010
S -2.80435337 -2.80435337 -8.41306010
S -8.41306010 -8.41306010 -8.41306010
Pb 2.80435337 14.02176684 -2.80435337
Pb 14.02176684 2.80435337 -2.80435337
Pb 8.41306010 8.41306010 -2.80435337
S -2.80435337 14.02176684 -2.80435337
Pb 2.80435337 2.80435337 -2.80435337
S 8.41306010 2.80435337 -2.80435337
S 2.80435337 8.41306010 -2.80435337
Pb -2.80435337 8.41306010 -2.80435337
Pb -8.41306010 2.80435337 -2.80435337
S -2.80435337 2.80435337 -2.80435337
S -8.41306010 8.41306010 -2.80435337
S -14.02176684 2.80435337 -2.80435337
S 14.02176684 -2.80435337 -2.80435337
Pb 8.41306010 -2.80435337 -2.80435337
Pb 2.80435337 -8.41306010 -2.80435337
S 8.41306010 -8.41306010 -2.80435337
S 2.80435337 -2.80435337 -2.80435337
Pb -2.80435337 -2.80435337 -2.80435337
Pb -8.41306010 -8.41306010 -2.80435337
S -2.80435337 -8.41306010 -2.80435337
S -8.41306010 -2.80435337 -2.80435337
Pb -14.02176684 -2.80435337 -2.80435337
S 2.80435337 -14.02176684 -2.80435337
Pb -2.80435337 -14.02176684 -2.80435337
S 2.80435337 14.02176684 2.80435337
S 14.02176684 2.80435337 2.80435337
S 8.41306010 8.41306010 2.80435337
Pb -2.80435337 14.02176684 2.80435337
Pb 2.80435337 8.41306010 2.80435337
Pb 8.41306010 2.80435337 2.80435337
S 2.80435337 2.80435337 2.80435337
Pb -8.41306010 8.41306010 2.80435337
Pb -2.80435337 2.80435337 2.80435337
```

```
S -2.80435337 8.41306010 2.80435337
S -8.41306010 2.80435337 2.80435337
Pb -14.02176684 2.80435337 2.80435337
Pb 14.02176684 -2.80435337 2.80435337
Pb 2.80435337 -2.80435337 2.80435337
Pb 8.41306010 -8.41306010 2.80435337
S 8.41306010 -2.80435337 2.80435337
S 2.80435337 -8.41306010 2.80435337
Pb -8.41306010 -2.80435337 2.80435337
Pb -2.80435337 -8.41306010 2.80435337
S -2.80435337 -2.80435337 2.80435337
S -8.41306010 -8.41306010 2.80435337
S -14.02176684 -2.80435337 2.80435337
Pb 2.80435337 -14.02176684 2.80435337
S -2.80435337 -14.02176684 2.80435337
Pb 8.41306010 8.41306010 8.41306010
Pb 2.80435337 2.80435337 8.41306010
S 8.41306010 2.80435337 8.41306010
S 2.80435337 8.41306010 8.41306010
Pb -2.80435337 8.41306010 8.41306010
Pb -8.41306010 2.80435337 8.41306010
S -2.80435337 2.80435337 8.41306010
S -8.41306010 8.41306010 8.41306010
Pb 8.41306010 -2.80435337 8.41306010
Pb 2.80435337 -8.41306010 8.41306010
S 8.41306010 -8.41306010 8.41306010
S 2.80435337 -2.80435337 8.41306010
Pb -2.80435337 -2.80435337 8.41306010
Pb -8.41306010 -8.41306010 8.41306010
S -2.80435337 -8.41306010 8.41306010
S -8.41306010 -2.80435337 8.41306010
S 2.80435337 2.80435337 14.02176684
Pb -2.80435337 2.80435337 14.02176684
Pb 2.80435337 -2.80435337 14.02176684
S -2.80435337 -2.80435337 14.02176684
```

## 16 Cartesian coordinates of $Pb_{140}S_{140}$ in atomic units

```
atom      x              y               z
Pb -19.63047357 2.80435337 8.41306010
Pb -19.63047357 8.41306010 2.80435337
S -19.63047357 2.80435337 2.80435337
Pb -19.63047357 -2.80435337 2.80435337
S -19.63047357 -2.80435337 8.41306010
S -19.63047357 -8.41306010 2.80435337
Pb -19.63047357 2.80435337 -2.80435337
S -19.63047357 8.41306010 -2.80435337
S -19.63047357 2.80435337 -8.41306010
Pb -19.63047357 -8.41306010 -2.80435337
Pb -19.63047357 -2.80435337 -8.41306010
S -19.63047357 -2.80435337 -2.80435337
Pb -14.02176684 2.80435337 14.02176684
S -14.02176684 8.41306010 14.02176684
Pb -14.02176684 -8.41306010 14.02176684
S -14.02176684 -2.80435337 14.02176684
Pb -14.02176684 14.02176684 2.80435337
S -14.02176684 14.02176684 8.41306010
Pb -14.02176684 8.41306010 8.41306010
Pb -14.02176684 2.80435337 2.80435337
S -14.02176684 2.80435337 8.41306010
S -14.02176684 8.41306010 2.80435337
Pb -14.02176684 -2.80435337 8.41306010
Pb -14.02176684 -8.41306010 2.80435337
S -14.02176684 -8.41306010 8.41306010
S -14.02176684 -2.80435337 2.80435337
Pb -14.02176684 -14.02176684 8.41306010
S -14.02176684 -14.02176684 2.80435337
Pb -14.02176684 14.02176684 -8.41306010
S -14.02176684 14.02176684 -2.80435337
Pb -14.02176684 8.41306010 -2.80435337
Pb -14.02176684 2.80435337 -8.41306010
S -14.02176684 2.80435337 -2.80435337
S -14.02176684 8.41306010 -8.41306010
Pb -14.02176684 -2.80435337 -2.80435337
Pb -14.02176684 -8.41306010 -8.41306010
S -14.02176684 -8.41306010 -2.80435337
S -14.02176684 -2.80435337 -8.41306010
Pb -14.02176684 -14.02176684 -2.80435337
S -14.02176684 -14.02176684 -8.41306010
Pb -14.02176684 8.41306010 -14.02176684
S -14.02176684 2.80435337 -14.02176684
Pb -14.02176684 -2.80435337 -14.02176684
S -14.02176684 -8.41306010 -14.02176684
Pb -8.41306010 -2.80435337 14.02176684
S -8.41306010 -8.41306010 14.02176684
Pb -8.41306010 -14.02176684 14.02176684
Pb -8.41306010 2.80435337 8.41306010
Pb -8.41306010 8.41306010 2.80435337
S -8.41306010 2.80435337 2.80435337
Pb -8.41306010 -8.41306010 8.41306010
Pb -8.41306010 -2.80435337 2.80435337
S -8.41306010 -2.80435337 8.41306010
```

S -8.41306010 -8.41306010 2.80435337
Pb -8.41306010 -14.02176684 2.80435337
S -8.41306010 -14.02176684 8.41306010
S -8.41306010 -19.63047357 2.80435337
Pb -8.41306010 14.02176684 -2.80435337
S -8.41306010 14.02176684 -8.41306010
Pb -8.41306010 2.80435337 -2.80435337
Pb -8.41306010 8.41306010 -8.41306010
S -8.41306010 8.41306010 -2.80435337
S -8.41306010 2.80435337 -8.41306010
Pb -8.41306010 -8.41306010 -2.80435337
Pb -8.41306010 -2.80435337 -8.41306010
S -8.41306010 -2.80435337 -2.80435337
S -8.41306010 -8.41306010 -8.41306010
Pb -8.41306010 -19.63047357 -2.80435337
Pb -8.41306010 -14.02176684 -8.41306010
S -8.41306010 -14.02176684 -2.80435337
Pb -8.41306010 14.02176684 -14.02176684
Pb -8.41306010 2.80435337 -14.02176684
S -8.41306010 8.41306010 -14.02176684
S -8.41306010 2.80435337 -19.63047357
Pb -8.41306010 -8.41306010 -14.02176684
Pb -8.41306010 -2.80435337 -19.63047357
S -8.41306010 -2.80435337 -14.02176684
S -8.41306010 -14.02176684 -14.02176684
S -8.41306010 14.02176684 14.02176684
Pb -8.41306010 2.80435337 19.63047357
Pb -8.41306010 8.41306010 14.02176684
S -8.41306010 2.80435337 14.02176684
S -8.41306010 -2.80435337 19.63047357
Pb -8.41306010 14.02176684 8.41306010
Pb -8.41306010 19.63047357 2.80435337
S -8.41306010 14.02176684 2.80435337
S -8.41306010 8.41306010 8.41306010
S -8.41306010 19.63047357 -2.80435337
Pb -2.80435337 -8.41306010 14.02176684
S -2.80435337 -8.41306010 19.63047357
S -2.80435337 -2.80435337 14.02176684
S -2.80435337 -14.02176684 14.02176684
Pb -2.80435337 2.80435337 2.80435337
S -2.80435337 2.80435337 8.41306010
S -2.80435337 8.41306010 2.80435337
Pb -2.80435337 -2.80435337 8.41306010
Pb -2.80435337 -8.41306010 2.80435337
S -2.80435337 -8.41306010 8.41306010
S -2.80435337 -2.80435337 2.80435337
Pb -2.80435337 -14.02176684 8.41306010
Pb -2.80435337 -19.63047357 2.80435337
S -2.80435337 -19.63047357 8.41306010
S -2.80435337 -14.02176684 2.80435337
Pb -2.80435337 14.02176684 -8.41306010
S -2.80435337 14.02176684 -2.80435337
S -2.80435337 19.63047357 -8.41306010
Pb -2.80435337 8.41306010 -2.80435337
Pb -2.80435337 2.80435337 -8.41306010

S -2.80435337 2.80435337 -2.80435337
S -2.80435337 8.41306010 -8.41306010
Pb -2.80435337 -2.80435337 -2.80435337
Pb -2.80435337 -8.41306010 -8.41306010
S -2.80435337 -8.41306010 -2.80435337
S -2.80435337 -2.80435337 -8.41306010
Pb -2.80435337 -14.02176684 -2.80435337
Pb -2.80435337 -19.63047357 -8.41306010
S -2.80435337 -19.63047357 -2.80435337
S -2.80435337 -14.02176684 -8.41306010
S -2.80435337 14.02176684 -14.02176684
Pb -2.80435337 8.41306010 -14.02176684
Pb -2.80435337 2.80435337 -19.63047357
S -2.80435337 2.80435337 -14.02176684
S -2.80435337 8.41306010 -19.63047357
Pb -2.80435337 -2.80435337 -14.02176684
Pb -2.80435337 -8.41306010 -19.63047357
S -2.80435337 -8.41306010 -14.02176684
S -2.80435337 -2.80435337 -19.63047357
Pb -2.80435337 -14.02176684 -14.02176684
Pb -2.80435337 14.02176684 14.02176684
Pb -2.80435337 8.41306010 19.63047357
Pb -2.80435337 2.80435337 14.02176684
S -2.80435337 2.80435337 19.63047357
S -2.80435337 8.41306010 14.02176684
Pb -2.80435337 -2.80435337 19.63047357
Pb -2.80435337 19.63047357 8.41306010
Pb -2.80435337 14.02176684 2.80435337
S -2.80435337 14.02176684 8.41306010
S -2.80435337 19.63047357 2.80435337
Pb -2.80435337 8.41306010 8.41306010
Pb -2.80435337 19.63047357 -2.80435337
Pb 2.80435337 -8.41306010 19.63047357
Pb 2.80435337 -2.80435337 14.02176684
S 2.80435337 -8.41306010 14.02176684
Pb 2.80435337 -14.02176684 14.02176684
Pb 2.80435337 2.80435337 8.41306010
Pb 2.80435337 8.41306010 2.80435337
S 2.80435337 2.80435337 2.80435337
Pb 2.80435337 -8.41306010 8.41306010
Pb 2.80435337 -2.80435337 2.80435337
S 2.80435337 -2.80435337 8.41306010
S 2.80435337 -8.41306010 2.80435337
Pb 2.80435337 -19.63047357 8.41306010
Pb 2.80435337 -14.02176684 2.80435337
S 2.80435337 -14.02176684 8.41306010
S 2.80435337 -19.63047357 2.80435337
Pb 2.80435337 14.02176684 -2.80435337
Pb 2.80435337 19.63047357 -8.41306010
S 2.80435337 14.02176684 -8.41306010
Pb 2.80435337 2.80435337 -2.80435337
Pb 2.80435337 8.41306010 -8.41306010
S 2.80435337 8.41306010 -2.80435337
S 2.80435337 2.80435337 -8.41306010
Pb 2.80435337 -8.41306010 -2.80435337

Pb 2.80435337 -2.80435337 -8.41306010
S 2.80435337 -2.80435337 -2.80435337
S 2.80435337 -8.41306010 -8.41306010
Pb 2.80435337 -19.63047357 -2.80435337
Pb 2.80435337 -14.02176684 -8.41306010
S 2.80435337 -14.02176684 -2.80435337
S 2.80435337 -19.63047357 -8.41306010
Pb 2.80435337 14.02176684 -14.02176684
Pb 2.80435337 2.80435337 -14.02176684
Pb 2.80435337 8.41306010 -19.63047357
S 2.80435337 8.41306010 -14.02176684
S 2.80435337 2.80435337 -19.63047357
Pb 2.80435337 -8.41306010 -14.02176684
Pb 2.80435337 -2.80435337 -19.63047357
S 2.80435337 -2.80435337 -14.02176684
S 2.80435337 -8.41306010 -19.63047357
S 2.80435337 -14.02176684 -14.02176684
S 2.80435337 14.02176684 14.02176684
Pb 2.80435337 2.80435337 19.63047357
Pb 2.80435337 8.41306010 14.02176684
S 2.80435337 8.41306010 19.63047357
S 2.80435337 2.80435337 14.02176684
S 2.80435337 -2.80435337 19.63047357
Pb 2.80435337 14.02176684 8.41306010
Pb 2.80435337 19.63047357 2.80435337
S 2.80435337 19.63047357 8.41306010
S 2.80435337 14.02176684 2.80435337
S 2.80435337 8.41306010 8.41306010
S 2.80435337 19.63047357 -2.80435337
Pb 8.41306010 -8.41306010 14.02176684
S 8.41306010 -2.80435337 14.02176684
S 8.41306010 -14.02176684 14.02176684
Pb 8.41306010 2.80435337 2.80435337
S 8.41306010 2.80435337 8.41306010
S 8.41306010 8.41306010 2.80435337
Pb 8.41306010 -2.80435337 8.41306010
Pb 8.41306010 -8.41306010 2.80435337
S 8.41306010 -8.41306010 8.41306010
S 8.41306010 -2.80435337 2.80435337
Pb 8.41306010 -14.02176684 8.41306010
Pb 8.41306010 -19.63047357 2.80435337
S 8.41306010 -14.02176684 2.80435337
Pb 8.41306010 14.02176684 -8.41306010
S 8.41306010 14.02176684 -2.80435337
Pb 8.41306010 8.41306010 -2.80435337
Pb 8.41306010 2.80435337 -8.41306010
S 8.41306010 2.80435337 -2.80435337
S 8.41306010 8.41306010 -8.41306010
Pb 8.41306010 -2.80435337 -2.80435337
Pb 8.41306010 -8.41306010 -8.41306010
S 8.41306010 -8.41306010 -2.80435337
S 8.41306010 -2.80435337 -8.41306010
Pb 8.41306010 -14.02176684 -2.80435337
S 8.41306010 -19.63047357 -2.80435337
S 8.41306010 -14.02176684 -8.41306010

S 8.41306010 14.02176684 -14.02176684
Pb 8.41306010 8.41306010 -14.02176684
Pb 8.41306010 2.80435337 -19.63047357
S 8.41306010 2.80435337 -14.02176684
Pb 8.41306010 -2.80435337 -14.02176684
S 8.41306010 -8.41306010 -14.02176684
S 8.41306010 -2.80435337 -19.63047357
Pb 8.41306010 -14.02176684 -14.02176684
Pb 8.41306010 14.02176684 14.02176684
Pb 8.41306010 2.80435337 14.02176684
S 8.41306010 2.80435337 19.63047357
S 8.41306010 8.41306010 14.02176684
Pb 8.41306010 -2.80435337 19.63047357
Pb 8.41306010 14.02176684 2.80435337
S 8.41306010 14.02176684 8.41306010
S 8.41306010 19.63047357 2.80435337
Pb 8.41306010 8.41306010 8.41306010
Pb 8.41306010 19.63047357 -2.80435337
Pb 14.02176684 8.41306010 14.02176684
S 14.02176684 2.80435337 14.02176684
Pb 14.02176684 -2.80435337 14.02176684
S 14.02176684 -8.41306010 14.02176684
Pb 14.02176684 14.02176684 8.41306010
S 14.02176684 14.02176684 2.80435337
Pb 14.02176684 2.80435337 8.41306010
Pb 14.02176684 8.41306010 2.80435337
S 14.02176684 8.41306010 8.41306010
S 14.02176684 2.80435337 2.80435337
Pb 14.02176684 -8.41306010 8.41306010
Pb 14.02176684 -2.80435337 2.80435337
S 14.02176684 -2.80435337 8.41306010
S 14.02176684 -8.41306010 2.80435337
Pb 14.02176684 -14.02176684 2.80435337
S 14.02176684 -14.02176684 8.41306010
Pb 14.02176684 14.02176684 -2.80435337
S 14.02176684 14.02176684 -8.41306010
Pb 14.02176684 2.80435337 -2.80435337
Pb 14.02176684 8.41306010 -8.41306010
S 14.02176684 8.41306010 -2.80435337
S 14.02176684 2.80435337 -8.41306010
Pb 14.02176684 -8.41306010 -2.80435337
Pb 14.02176684 -2.80435337 -8.41306010
S 14.02176684 -2.80435337 -2.80435337
S 14.02176684 -8.41306010 -8.41306010
Pb 14.02176684 -14.02176684 -8.41306010
S 14.02176684 -14.02176684 -2.80435337
Pb 14.02176684 2.80435337 -14.02176684
S 14.02176684 8.41306010 -14.02176684
Pb 14.02176684 -8.41306010 -14.02176684
S 14.02176684 -2.80435337 -14.02176684
Pb 19.63047357 2.80435337 2.80435337
S 19.63047357 2.80435337 8.41306010
S 19.63047357 8.41306010 2.80435337
Pb 19.63047357 -2.80435337 8.41306010
Pb 19.63047357 -8.41306010 2.80435337

S 19.63047357 -2.80435337 2.80435337
Pb 19.63047357 8.41306010 -2.80435337
Pb 19.63047357 2.80435337 -8.41306010
S 19.63047357 2.80435337 -2.80435337
Pb 19.63047357 -2.80435337 -2.80435337
S 19.63047357 -8.41306010 -2.80435337
S 19.63047357 -2.80435337 -8.41306010
