# Supplementary material for: Enhancement of Spontaneous Photon Emission in Inverse Photoemission Transitions in Semiconductor Quantum Dots
Source: J Phys Chem Lett. 2024 Jan 4;15(2):364–70. doi: 10.1021/acs.jpclett.3c02934 (PMC10801682; doi:10.1021/acs.jpclett.3c02934)
Supplement: Supplementary file 2 — jz3c02934_si_002.pdf [file jz3c02934_si_002.pdf]

jz-2023-02934s.R1

Name: Peer Review Information for "Enhancement of Spontaneous Photon Emission in Inverse Photoemission Transitions in Semiconductor Quantum Dots"

First Round of Reviewer Comments

Reviewer: 1

Comments to the Author

1. What is the major advance reported in the paper?

This manuscript investigates the effects of an external electric field on the inverse photoemission (IPE) in CdSe, CdS, PbSe and PbS quantum dots (QDs). The authors demonstrate that an external electric field can enhance IPE in QDs, using time-dependent perturbation theory, with the electronic structure characterized by DFT. The effects of the electric field on the emission intensities and frequencies are calculated. While the manuscript reports interesting results and the methodology used to describe the excited states of QDs is state-of-the-art, I am not convinced the manuscript should be published in JPCL. I feel that in the present form, it is more suitable for JPC C, JCP, or perhaps Phys Rev B. If the authors want to make this work more suitable for JPCL (or PRL), I suggest providing a detailed analysis of the field effects or/and making a connection with experimental measurements.

a) Why does a relatively small anisotropy of QDs result in a strong dependence of IPE on the field direction?

b) Why does the sign of frequency shift depend on the type of QD and field direction?

c) Is there any evidence of these field effects in experiments? If not, what experiment can be proposed to observe these effects?

2. What is the immediate significance of this advance?

This is not entirely clear to me. IPE has been known for a long time. While the authors' computational results demonstrate that the intensity and frequency of IPE can be controlled with an external field, it is hard to believe that this was not tried before. If the authors are convinced that an external electric field provides a novel way to control IPE, this should be clearly stated.

3. Technical suggestions

a) It is not clear to me how QDs are oriented with respect to the external electric field. The effects are described in terms of the field direction (x,y,z components), this is not very useful if the nanoparticle orientation is not known.

b) A reference to the software package (TERACHEM or TeraChem?) should be added.

c) Input files, or at least atomic coordinates of QDs should be provided in SI.

Reviewer: 2

Comments to the Author

In this paper the Authors used perturbation theory to examine the effects of external, electric fields on the inverse photoemission process in quantum dot systems. Their results show that specific field strengths, and field orientations, can selectively enhance IPS features in CdSe, CdS, PbSe, and Pbs quantum dots.

The subject of this paper is interesting, and applicable to The Journal of Physical Chemistry Letters, however there are several major questions that need to be answered before the manuscript is ready for publication.

Following are several comments and questions for the Authors to consider:

1. The structures used by the Authors are mentioned to come from bulk systems. Have their geometries been optimized in any way? Especially while under electric fields, do the Authors expect there to be any geometric changes that can influence the IPE spectra?

2. What are the approximate diameter and crystal structure of the QDs examined herein?

3. Have the QD surfaces been treated in any way to avoid spurious surface effects?

4. On page 5, equation 1, are the values integrated for the area under the peaks, or are these the maximum values of the transition probability? Are the Authors interested in or able to capture increased responses where the lines are broadened?

5. On page 5, equation2, do the authors ensure that the response is the same character when the field is on vs. when the field is off. This comes up (for example) on page 6, Table 2 where the authors claim there is a dw of 0.8 eV for the Cd45S45 spectrum, except, looking at Figure S6 A, the spectra appear to be the same, with minor differences to the intensity of the peaks. This seems like less a spectral shift (e.g., the first absorption peak blueshifting with decreasing system size in a quantum dot) and seems to be that select transitions are now more favored due to changes in symmetry or molecular orbital splitting. Can the authors comment on their justification for this definition of stark shift?

6. A) On page 5, Table 2 are listed enhancement values for the quantum dot systems under fields of various orientations. What field strengths do these values correspond to (E1,E2,E4,E5 as defined on lines 47 and 48 of page 5)? B) I am surprised by the large differences the orientation of the field makes, especially since going off Fig. 1 (page 3) these quantum dots are roughly spherical. I would have expected that for various field directions (in x, y, and z) the change would be similar (like the Cd45S45 and Pb44S44 systems), however for some systems (most notably Cd24S24, Pb140S140, and Pb29Se29) there appear to be large variations of the transition probabilities with field direction. Can the Authors discuss what gives rise to these large differences? C) Furthermore, the CdS, CdSe, and PbS systems all seem to show an enhancement with system size; however, the PbSe system has a large decrease with increasing system size. Do the Authors expect there to be a trend between system size and observed enhancement, and, if so, what separates the PbSe system?

7. I am surprised by the field-dependent spectra (Figure 4, page 6), in that I would anticipate increasing the electric field would lead to select transitions being enhanced and shifted (as appears to be the case in Figure S5). This behavior is apparent for the E3 to E5 series in Figure 4, can the authors discuss what happens between E2 and E1 to cause the appearance of several transitions not seen in the other electric fields, and the apparent breaking of the trend where the transition probability increases with field strength?

8. In the text (Page 6, lines 40-44) it is mentioned that the E2 spectra is a blue shift (as noted in Table 2), but that E4 and E5 do not show similar shifting behavior. This raises three questions:

A) This blue-shifted transition appears to be at the same energy as the third transition in the zero-field case, how do the authors identify that the character of the peak changes between E3 and E2?

B) In the E2 energy plot, where do the ~3.75 eV and ~4.0 eV transitions go, are they under the peak or are they shifted elsewhere in the spectrum?

C) Why do the authors suspect that the E4 and E5 peak positions are unaffected by the applied field?

9. On page 6, Table 2, what field strength (E1,2,4,5) do the field-on shift values correspond to?

10. On page 7, Table 3, are all the QD systems oriented the same way? The field direction definitions appear to be external (page 10, lines 10-15), can these be related to crystal indices (such as a Miller index)? I ask this as it is odd to me that the same crystal structure and material could have different maximally coupled field directions. Are these QDs different sizes of the same bulk crystal phase? Can the maximally coupled field direction be predicted for different systems (e.g., a larger CdSe dot) based on information obtained from the smaller QDs?

11. Do the authors think that their initial Hartree-Fock method is able to provide a good initial wavefunction for their perturbation theory? Are there any experimental justifications? Can the authors provide the band gaps for all the systems? I ask as the only provided band gaps are for PbS (large and small) and CdSe, and while CdSe's value of 1.8 eV seems to match with the expected value of ~1.7 eV for bulk; the PbS band gap (5.6 eV for the larger system) does not agree with the anticipated value of ~0.5-2.5 eV (https://doi.org/10.1021/acsnano.5b06833, ref. 12 in the text).

12. Page 10, line 39, the 'section' reference is missing the section number.

Reviewer: 3

Comments to the Author

The manuscript "Enhancement of spontaneous photon emission in inverse photoemission transitions in semiconductor quantum dots" Spanedda, Mesta, and Chakraborty discuss the enhancement of inverse photoemission using electric fields. The work is interesting and potentially applicable to enhancing experimental methods.

Minor Comments

(1) Heuristic Picture: Is there a heuristic picture for why the electric field enhances the photon emission by as much as it does?

(2) Entanglement with an Electric Field: Is (1) potentially related to the ability to modulate entanglement as discussed in this paper "Entangling and disentangling many-electron quantum systems with an electric field" (https://doi.org/10.1103/PhysRevA.97.062502)?

(3) Geminal Method: Can the authors add a few additional sentences about the geminal part of the calculation prior to TDDFT? It would be helpful to have this information here for completeness even if it is elsewhere in the literature.

In summary, after the authors address the above questions, I recommend that the manuscript be accepted for publication.

Author's Response to Peer Review Comments:

Dear Editor,

Thank you for sharing the reviewers comment with us. We have addressed all the editorial and reviewers' comments and our detailed responds is attached as a PDF file.

Sincerely

Ari Chakraborty

Response to the comments about the submitted paper: We thank the editor and the reviewers for their constructive comments. We have addressed all of them and modified the paper accordingly. Our detailed answers to the questions are presented in this document. Please note that reviewers' comments are in blue while our responses are in black.

Answers to Editor

Comment Editor.1 Supporting Information Statement: A brief, nonsentence description of the actual contents of each supporting information file is required. This description should be labeled Supporting Information and should appear before the Acknowledgement and Reference sections. Examples of sufficient and insufficient descriptions are as follows: *Examples of sufficient descriptions: "Supporting Information: 1H NMR spectra for all compounds" or "Additional experimental details, materials, and methods, including photographs of experimental setup". *Examples of insufficient descriptions: "Supporting Information: Figures S1-S3" or "Additional figures as mentioned in the text".

Answer to Editor.1 We have added the following statement in the revised manuscript:

Additional details including (1) derivation of the IPE transition probability, (2) IPE spectra for the zero-field cases, (3) effect of direction of IPE transition propabality, (4) enhancement of field-matter interaction, (5) list of electric field directions, (6) diameters of the QD, (7) HOMO-LUMO gap of the QDs, (8) derivation of Stark-field induced mixing of molecular orbitals, and (9) list of atomic coordinates of QDs are presented in the supporting information.

Comment Editor.2 Title: In both the main manuscript file and the Supporting Information, set the title in title case, with the first letter of each principal word capitalized.

Answer to Editor.2 We have made these changes in the revised manuscript.

Comment Editor.3 Supporting Information: Please add full header at top of page of the Supporting Information file, which includes: Title, Full Author List, and Author affiliations (exactly as they appear in the manuscript).

Answer to Editor.3 We have made these changes in the revised manuscript.

Comment Editor.4 Title and Author Lists: Title, author names, and affiliations must match in three places: (1) manuscript file, (2) supporting information, and (3) ACS Paragon Plus.

Answer to Editor.4 We have made these changes in the revised manuscript.

Comment Editor.5 References: In both the main file and the supporting information, fix the style of all references to use JPCL formatting (check all references carefully). ***JPC Letters reference formatting requires that journal references

should contain: () around numbers; author names; article title (titles entirely in title case or entirely in lower case); abbreviated journal title (italicized); year (bolded); volume (italicized); and pages (first-last). Book references should contain author names; book title (in the same pattern); publisher; city; and year. Websites must include date of access.
Answer to Editor.5 We have made these changes in the revised manuscript.

Comment Editor.6 Supporting Information: Please number SI pages in the following format: "S1, S2..."
Answer to Editor.6 We have made these changes in the revised manuscript.

Answers to Reviewer 1

Comment R1.1 If the authors want to make this work more suitable for JPCL (or PRL), I suggest providing a detailed analysis of the field effects or/and making a connection with experimental measurements.

Answer to R1.1 The manuscript has been revised to include references that discuss the use of directed electric fields and their potential applications in chemical catalysis and reaction control. The added references include:

- Electric-Field Mediated Chemistry: Uncovering and Exploiting the Potential of (Oriented) Electric Fields to Exert Chemical Catalysis and Reaction Control (JACS 2020)
- Oriented electric fields as future smart reagents in chemistry (Nature 2016)
- Emission properties and temporal coherence of the dark exciton confined in a GaAs quantum dot (PRB 2021)
- Optical Stark shift to control the dark exciton occupation of a quantum dot in a tilted magnetic field (PRB 2021)
- Dressed-State Resonant Coupling between Bright and Dark Spins in Diamond (PRL 2013)
- Dark-State Luminescence of Macroatoms at the Near Field (PRL 2005)
- Radiative control of dark excitons at room temperature by nano-optical antenna-tip Purcell effect (Nature 2018)
- Electrostatic Field-Induced Oscillator Strength Focusing in Molecules (ACS JPCB 2020)

Furthermore, a new subsection titled *Stark field enhancement is a consequence of orbital mixing* has been added to provide additional analysis of the results. Additionally, a new section in the supporting information has been included, titled *Derivation of Stark-field induced mixing of molecular orbitals* which presents the derivation of the results discussed in the main manuscript.

Comment R1.2 Why does a relatively small anisotropy of QDs result in a strong dependence of IPE on the field direction

Answer to R1.2 The strong dependence of spectra on the field direction in quantum dots is primarily due to the spatial characteristics of the molecular orbitals involved in the spontaneous emission process. While the quantum dot may have

an almost spherical shape, the dominant molecular orbitals responsible for the transitions may not be s-type orbitals and can exhibit angular dependence. In other words, the spherical nature of the quantum dot does not imply that all the molecular orbitals within it are also spherical.

Comment R1.3 Why does the sign of frequency shift depend on the type of QD and field direction?
Answer to R1.3 The frequency shift depends on various factors, such as the energy of the transition, matrix elements of the dipole operator, and the spatial representation of the molecular orbitals (MO) in both the field-on and field-off cases. Currently, there is no known model capable of predicting the sign of the frequency shift without performing the quantum mechanical calculations first. Therefore, we believe that the only reliable way to determine the sign is by conducting the necessary QM calculations.

Comment R1.4 Is there any evidence of these field effects in experiments?
Answer to R1.4 The quantum confined Stark effect in quantum dots has been extensively studied and experimentally observed in photoluminescence processes. In the revised manuscript, we have included references 22-23 to support these findings. However, the investigation of the Stark effect for IPE (Inverse Photoemission) is relatively new, and our literature survey indicates that it has not been previously explored. This highlights the novelty of our research in exploring the Stark effect in the context of IPE.

Comment R1.5 If not, what experiment can be proposed to observe these effects?
Answer to R1.5 We anticipate that conducting IPE experiments under weak external electric fields will result in an enhancement of photon emission. We believe that the outcomes of this research will serve as motivation for further experimental investigations into these materials.

Comment R1.6 IPE has been known for a long time. While the authors' computational results demonstrate that the intensity and frequency of IPE can be controlled with an external field, it is hard to believe that this was not tried before. If the authors are convinced that an external electric field provides a novel way to control IPE, this should be clearly stated.
Answer to R1.6 While both the IPE and Stark effect have been known for a significant period, the combined application of IPE and the orientated Stark effect

in quantum dots (QDs) has not been extensively studied. This research provides precise predictions regarding the level of enhancement achievable for various QDs when subjected to weak directed Stark fields. Furthermore, this study introduces a novel theoretical and computational approach for investigating field-enhanced IPE spectra. Calculating IPE spectra poses challenges and exceeds the capabilities of both commercial and noncommercial electronic structure packages. This work presents two distinct approaches (MBPT and FD-GSIK) for calculating IPE spectra in QDs, enhancing the computational toolkit available to both experimental and computational material scientists.

Comment R1.7 It is not clear to me how QDs are oriented with respect to the external electric field.
Answer to R1.7 In all cases, the QD is placed at the origin and the field-directions are along the Cartesian directions.

Comment R1.8 A reference to the software package (TERACHEM or TeraChem?) should be added.
Answer to R1.8 We have added the references to TERACHEM in the revised manuscript.

Comment R1.9 Input files, or at least atomic coordinates of QDs should be provided in SI.
Answer to R1.9 We have added the atomic coordinates of the QDs in the SI.

Answers to Reviewer 2

Comment R2.1 The structures used by the Authors are mentioned to come from bulk systems. Have their geometries been optimized in any way? Especially while under electric fields, do the Authors expect there to be any geometric changes that can influence the IPE spectra?

Answer to R2.1 The geometries of the quantum dots (QDs) were not optimized and were intentionally maintained on the bulk structures. This approach enables a systematic evaluation of the impact of adding atoms to the QDs. The calculations were conducted under the weak-field limit, suggesting that any changes in geometry are expected to be minimal. However, we acknowledge that non-zero temperature can lead to significant structural distortions. Therefore, as the next phase of this study, we intend to perform ensemble-averaged calculations using a canonical distribution of geometries to account for the influence of temperature on the QD structures.

Comment R2.2 What are the approximate diameter and crystal structure of the QDs examined herein?

Answer to R2.2 We have added information about the dot diameters in Table. 3 of the revised manuscript.

Comment R2.3 On page 5, equation 1, are the values integrated for the area under the peaks, or are these the maximum values of the transition probability?

Answer to R2.3 The values are ratios of the maximum values of transition probability. We also calculated the integrated form which is presented in Eq. 3

Comment R2.4 Are the Authors interested in or able to capture increased responses where the lines are broadened?

Answer to R2.4 We are indeed interested in studying the overall response of the system, and we evaluate this response by integrating the squared difference of the entire spectra across the frequency range. The mathematical expression for quantifying the change in the overall spectra is presented in Eq. 3, and the results are presented in Table. 3 of the revised manuscript.

Comment R2.5 On page 5, Table 2 are listed enhancement values for the quantum dot systems under fields of various orientations. What field strengths do these values correspond to (E1,E2,E4,E5 as defined on lines 47 and 48 of page 5)?

Answer to R2.5 We have added the information of the field strengths in Table. 1 of Supporting Information

Comment R2.6 I am surprised by the large differences the orientation of the field makes, especially since going off Fig. 1 (page 3) these quantum dots are roughly spherical. I would have expected that for various field directions (in x, y, and z) the change would be similar (like the Cd45S45 and Pb44S44 systems), however for some systems (most notably Cd24S24, Pb140S140, and Pb29Se29) there appear to be large variations of the transition probabilities with field direction. Can the Authors discuss what gives rise to these large differences?

Answer to R2.6 The influence of direction arises from the intricate angular dependence of molecular orbitals involved in electronic transitions. Similar to atoms, spherical quantum dots possess not only s-type orbitals but also p, d, and other higher angular momentum orbitals. Due to the non-spherical nature of these orbitals, their response to the applied electric field varies with direction. To put it differently, the spherical characteristic of the quantum dot does not preclude the involvement of other higher-order spherical harmonic interaction terms from its molecular orbitals.

Comment R2.7 Furthermore, the CdS, CdSe, and PbS systems all seem to show an enhancement with system size; however, the PbSe system has a large decrease with increasing system size. Do the Authors expect there to be a trend between system size and observed enhancement, and, if so, what separates the PbSe system?

Answer to R2.7 The reviewer's comment regarding the decrease in the PbSe system with increasing size, as presented in Table 1, is correct. However, it is important to note that the fields in Table 1 were aligned solely along the Cartesian (x, y, z) directions. Upon optimizing the field directions, we discovered that the maximum enhancement for PbSe QDs can be significantly improved. The findings in Table 3 highlight the field directions that yield the highest enhancement for PbSe QDs. We plan to explore the relationship between QD size and enhancement propensity as the next step. However, to comprehensively investigate size-dependent scaling, we will require additional data points (currently limited to two per system) in future studies.

Comment R2.8 I am surprised by the field-dependent spectra (Figure 4, page 6), in that I would anticipate increasing the electric field would lead to select transitions being enhanced and shifted (as appears to be the case in Figure S5). This behavior is apparent for the E3 to E5 series in Figure 4, can the authors discuss what happens between E2 and E1 to cause the appearance of several

transitions not seen in the other electric fields, and the apparent breaking of the trend where the transition probability increases with field strength?

Answer to R2.8 The reviewer has raised a very important point and we have added a new section titled *Stark field enhancement is a consequence of orbital mixing* in the revised manuscript to discuss this point. The presence of the Stark field results in mixing of field-free molecular orbitals (MO). In the field-off case, the Fock operator $F^{(E=0)}$ is diagonal in the MO basis. The presence of the field adds a new interaction term and the field-on Fock operator $F^{(E)}$. In block-matrix notation is given as,

$$\boldsymbol{F}^{(E)} = \begin{bmatrix} \boldsymbol{\epsilon}_{\mathrm{o}} & \mathbf{0} \\ \mathbf{0} & \boldsymbol{\epsilon}_{\mathrm{v}} \end{bmatrix} + \begin{bmatrix} \boldsymbol{V}_{\mathrm{oo}}^{(\mathrm{E})} & \boldsymbol{V}_{\mathrm{ov}}^{(\mathrm{E})} \\ \boldsymbol{V}_{\mathrm{vo}}^{(\mathrm{E})} & \boldsymbol{V}_{\mathrm{vv}}^{(\mathrm{E})} \end{bmatrix} \tag{1}$$

where, o and v represents occupied and virtual MOs. Diagonalization of the $F^{(E)}$ generate the new field-on orbital energies and wavefunction $\{\epsilon_p^{(E)}, \chi_p^{(E)}\}$,

$$\boldsymbol{U}^{\dagger}\boldsymbol{F}^{(E)}\boldsymbol{U} = \begin{bmatrix} \boldsymbol{\epsilon}_{\mathrm{o}}^{(E)} & \mathbf{0} \\ \mathbf{0} & \boldsymbol{\epsilon}_{\mathrm{v}}^{(E)} \end{bmatrix} \tag{2}$$

where unitary matrix $\boldsymbol{U}$ diagonalizes the field-on Fock matrix. We note that $\boldsymbol{U}$ is field dependent

$$\boldsymbol{U}^{\dagger}\boldsymbol{U} = \boldsymbol{U}_E^{\dagger}\boldsymbol{U}_E = \boldsymbol{I} \tag{3}$$

and the column vectors of U are the mixing coefficient for the field-on MOs.

$$\chi_p^{(E)} = \sum_{k=1}^{N_{\mathrm{o}}} U_{kp}\chi_k^{(E=0)} + \sum_{c=1}^{N_{\mathrm{v}}} U_{cp}\chi_c^{(E=0)} \tag{4}$$

The dipole-moment operator μ is responsible for light-matter interaction and is also impacted by the presence of the Stark field. The matrix elements of the field-on dipole-moment operator can be obtained from field-off operators from the following unitary transformation.

$$\boldsymbol{\mu}^{(E)} = \boldsymbol{U}_E^{\dagger} \begin{bmatrix} \boldsymbol{\mu}_{\mathrm{oo}}^{(\mathrm{E=0})} & \boldsymbol{\mu}_{\mathrm{ov}}^{(\mathrm{E=0})} \\ \boldsymbol{\mu}_{\mathrm{vo}}^{(\mathrm{E=0})} & \boldsymbol{\mu}_{\mathrm{vv}}^{(\mathrm{E=0})} \end{bmatrix} \boldsymbol{U}_E \tag{5}$$

Analysis of the field-on virtual-virtual block of $\boldsymbol{\mu}$ which is responsible for intra-band transitions

$$\boldsymbol{\mu}_{\mathrm{vv}}^{(E)} = \boldsymbol{U}_{\mathrm{vv}}^{\dagger}\boldsymbol{\mu}_{\mathrm{vv}}^{(\mathrm{E=0})}\boldsymbol{U}_{\mathrm{vv}} + \boldsymbol{U}_{\mathrm{vo}}^{\dagger}\boldsymbol{\mu}_{\mathrm{oo}}^{(\mathrm{E=0})}\boldsymbol{U}_{\mathrm{ov}} + \boldsymbol{U}_{\mathrm{vo}}^{\dagger}\boldsymbol{\mu}_{\mathrm{ov}}^{(\mathrm{E=0})}\boldsymbol{U}_{\mathrm{vv}} + \boldsymbol{U}_{\mathrm{vv}}^{\dagger}\boldsymbol{\mu}_{\mathrm{vo}}^{(\mathrm{E=0})}\boldsymbol{U}_{\mathrm{ov}} \tag{6}$$

reveal that the field-on $\boldsymbol{\mu}_{\mathrm{vv}}^{(E)}$ block not only depends on the field-off virtual-virtual block, but also on field-off occupied-occupied and occupied-virtual blocks. The relative contribution of the four terms in Equation 6 determines which transition will dominate when the field is turned on. Because of the complex nature of the interaction, it is not possible for us to predict the relative importance of the terms without doing the calculation.

We have added a discussion on this point on page 8 and 9 of the revised manuscript. We have also summarized the contribution to the field-on diople operator using Feynman diagrams in Fig. 6. In addition to that, we have added an expanded form of the above derivation in the supporting information. Because of the mixing of orbitals, transitions that were dark in field-off case might become bright in the field-on case. We have added the following references that have demonstrated this effect in previous studies:

- Emission properties and temporal coherence of the dark exciton confined in a GaAs quantum dot (PRB 2021)
- Optical Stark shift to control the dark exciton occupation of a quantum dot in a tilted magnetic field (PRB 2021)
- Dressed-State Resonant Coupling between Bright and Dark Spins in Diamond (PRL 2013)
- Dark-State Luminescence of Macroatoms at the Near Field (PRL 2005)
- Radiative control of dark excitons at room temperature by nano-optical antenna-tip Purcell effect (Nature 2018)
- Electrostatic Field-Induced Oscillator Strength Focusing in Molecules (ACS JPCB 2020)

Comment R2.9 On page 6, Table 2, what field strength (E1,2,4,5) do the field-on shift values correspond to?
Answer to R2.9 We added this information in the table 2 of the revised manuscript.

Comment R2.10 Do the authors think that their initial Hartree-Fock method is able to provide a good initial wavefunction for their perturbation theory? Are there any experimental justifications?

Answer to R2.10 We consider Hartree-Fock (HF) to be a dependable method for providing an initial representation of the wavefunction. In this study, the HF wavefunction was utilized not only for perturbation theory calculations but also for the non-perturbative FDGSIK method. The fact that both of these distinct approaches (MBPT and FDGSIK) yield comparable results (as shown in Figure 1-3 in the supporting information) instills confidence in the stability of the HF wavefunction. However, it is important to note that the HF wavefunction lacks electron-electron correlation and is not suitable for direct comparison with experimental findings.

Comment R2.11 Can the authors provide the band gaps for all the systems? I ask as the only provided band gaps are for PbS (large and small) and CdSe, and while CdSe's value of 1.8 eV seems to match with the expected value of 1.7 eV for bulk; the PbS band gap (5.6 eV for the larger system) does not agree with the anticipated value of 0.5-2.5 eV (https://doi.org/10.1021/acsnano.5b06833, ref. 12 in the text).

Answer to R2.11 We have provided the HOMO-LUMO gap values in the supporting information (Table. 3).

The reviewer has brought up a significant and nuanced point. The disparity between the calculated HOMO-LUMO gap value of 5.6 eV from the HF calculation and the experimentally determined band gap (or quasiparticle gap) of $0.5 - 2.5$ eV arises from the difference in the definitions of these two quantities. The HOMO-LUMO gap obtained from the HF calculation does not account for electron-electron correlation, whereas the experimentally determined band gap does. To accurately compute the band gap, electron-electron correlation correction must be included, which can be achieved through DFT or the GW method. Sklenard and co-authors conducted a comprehensive analysis of the impact of electron-electron correlation on the band gap in PbS QDs in their article published in JPCL 2022.

- Size and Solvation Effects on Electronic and Optical Properties of PbS Quantum Dots $J.Phys.Chem.Lett.2022, 13, 90449050$

Fig.1 Panel:c of this article show that inclusion of electron-electron correlation decreases the band gap in the these QD and is needed to match experimental values. Becuause in this work we report only HOMO-LUMO gap (and not band gap) we have modified the discussion accordingly in the revised manuscript. We thank the reviewer for catching this subtle point and increasing the readability of the manuscript.

Comment R2.12 Page 10, line 39, the 'section' reference is missing the section number.
Answer to R2.12 We have corrected this error in the revised manuscript.

Answers to Reviewer 3

Comment R3.1 Heuristic Picture: Is there a heuristic picture for why the electric field enhances the photon emission by as much as it does?

Answer to R3.1 The phenomenon can be understood heuristically as the electric field inducing orbital mixing, causing previously dark transitions with low oscillator strengths to acquire oscillator strengths from the bright transitions. This effect of orbital mixing has been observed in various materials, and we have added the following references into the revised manuscript:

- Optical Stark shift to control the dark exciton occupation of a quantum dot in a tilted magnetic field (PRB 2021)
- Dressed-State Resonant Coupling between Bright and Dark Spins in Diamond (PRL 2013)
- Dark-State Luminescence of Macroatoms at the Near Field (PRL 2005)
- Electrostatic Field-Induced Oscillator Strength Focusing in Molecules (ACS JPCB 2020)

In order to provide a visual depiction that intuitively illustrates the influence of an electric field on the dipole-moment operator, we have included a Feynman diagram in the revised manuscript. This diagram effectively demonstrates that, in the presence of an electric field, the dipole-moment operator is influenced by four distinct terms, collectively resulting in an enhancement of the interaction between light and matter.

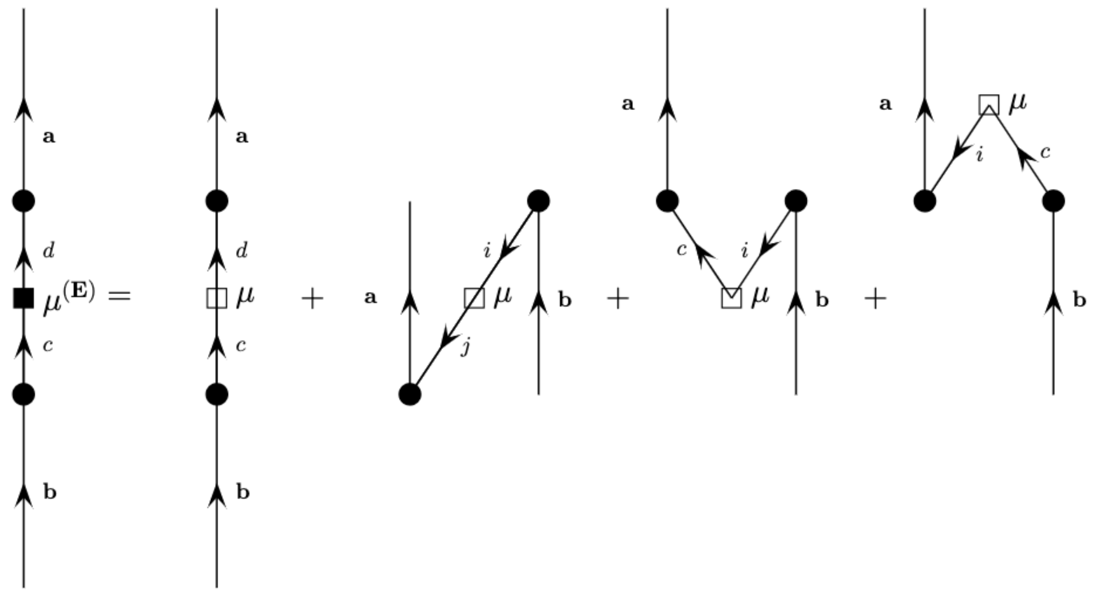

Figure R1: Feynman-Goldstone diagrams of the contributing terms to the field-dependent dipole-moment operator. The filled boxes and circles represent field-dependent quantities, while the unfilled boxes represent field-independent quantities.

Comment R3.2 Entanglement with an Electric Field: Is (1) potentially related to the ability to modulate entanglement as discussed in this paper "Entangling and disentangling many-electron quantum systems with an electric field" (https://doi.org/10.1103/PhysRevA.97.062502)?

Answer to R3.2 We thank the reviewer for bringing this significant article to our attention. The study presented in this article is relevant to our current work and we have included it in the revised manuscript.

Comment R3.3 Geminal Method: Can the authors add a few additional sentences about the geminal part of the calculation prior to TDDFT? It would be helpful to have this information here for completeness even if it is elsewhere in the literature.

Answer to R3.3 We have added a description of the FD-GSIK method and have summarized the key steps in the performing the calculations.
